# Enhanced NSAIDs Solubility in Drug–Drug Formulations with Ciprofloxacin †

**DOI:** 10.3390/ijms24043305

**Published:** 2023-02-07

**Authors:** Francisco Javier Acebedo-Martínez, Alicia Domínguez-Martín, Carolina Alarcón-Payer, Alejandro Sevillano-Páez, Cristóbal Verdugo-Escamilla, Josefa María González-Pérez, Fernando Martínez-Checa, Duane Choquesillo-Lazarte

**Affiliations:** 1 Laboratorio de Estudios Cristalográficos, IACT, CSIC-Universidad de Granada, Avda. de las Palmeras 4, 18100 Armilla, Spain; 2 Department of Inorganic Chemistry, Faculty of Pharmacy, University of Granada, 18071 Granada, Spain; 3 Servicio de Farmacia, Hospital Universitario Virgen de las Nieves, 18014 Granada, Spain; 4 Department of Microbiology, Faculty of Pharmacy, University of Granada, 18071 Granada, Spain; 5 Institute of Biotechnology, Biomedical Research Centre (CIBM), University of Granada, 18016 Granada, Spain

**Keywords:** NSAIDs, ciprofloxacin, molecular salts, mechanochemistry, crystal engineering

## Abstract

Drug–drug salts are a kind of pharmaceutical multicomponent solid in which the two co-existing components are active pharmaceutical ingredients (APIs) in their ionized forms. This novel approach has attracted great interest in the pharmaceutical industry since it not only allows concomitant formulations but also has proved potential to improve the pharmacokinetics of the involved APIs. This is especially interesting for those APIs that have relevant dose-dependent secondary effects, such as non-steroidal anti-inflammatory drugs (NSAIDs). In this work, six multidrug salts involving six different NSAIDs and the antibiotic ciprofloxacin are reported. The novel solids were synthesized using mechanochemical methods and comprehensively characterized in the solid state. Moreover, solubility and stability studies, as well as bacterial inhibition assays, were performed. Our results suggest that our drug–drug formulations enhanced the solubility of NSAIDs without affecting the antibiotic efficacy.

## 1. Introduction

Nearly 90% of the current drug pipeline suffers from different pharmacokinetic limitations, with low water solubility being the most relevant [1]. This is, in fact, a momentous problem for the pharmaceutical industry because it means a decrease in the oral bioavailability of active pharmaceutical ingredients (APIs). Advances in techniques to improve solubility are therefore becoming increasingly important, especially for oral-administration drugs. One of the most recent approaches in the industry is the formulation of multicomponent pharmaceutical solids, such as cocrystals and molecular salts. This strategy is rather interesting since it makes it possible to keep the pharmacological properties of already existing APIs intact while tailoring their physicochemical properties; i.e., melting point, solubility, thermodynamic stability, hygroscopicity, etc. [2].

Drug–drug salts are a special kind of multicomponent pharmaceutical solid in which all components are ionized APIs. Salt formation is the most common and effective method used to enhance the solubility and dissolution rate of acidic and basic drugs [3,4]. However, not all APIs can yield multidrug salts. Only those that show a significant p*K*_a_ difference between APIs lead to proton transfer; i.e., to the formation of salts [5]. The use of a combination of several APIs in a single formulation does not merely aim to join drugs to increase patients’ compliance but primarily seeks to improve the pharmacokinetics of the parent APIs, thus reducing side effects and improving efficacy by promoting synergic effects [2,4].

Non-steroidal anti-inflammatory drugs (NSAIDs) are one of the most consumed types of drugs worldwide. They are used to treat a wide range of conditions—mainly to relieve short- and long-term pain, reduce inflammation, and bring down fever—via the inhibition of the cyclooxygenase enzyme (COX). Nevertheless, NSAIDs are also associated with a broad spectrum of well-known secondary effects, mostly derived from their poor solubility, which are, therefore, dose-dependent side effects [6]. NSAIDs are often co-administered with other drugs. It should be noted that NSAIDs and antibiotics are concomitantly prescribed when infections are linked to inflammatory processes. Since this combination is rather common, it can be considered a good candidate for drug–drug multicomponent formulations. 

**Scheme 1:**
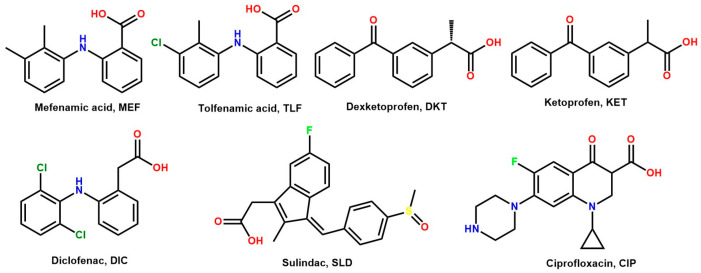
Chemical formulas for mefenamic acid (MEF), tolfenamic acid (TLF), dexketoprofen (DKT), ketoprofen (KET), diclofenac (DIC), sulindac (SLD), and ciprofloxacin (CIP).

Herein, the synthesis and characterization of six pharmaceutical drug–drug salts involving six different NSAIDs—mefenamic (MEF) and tolfenamic (TLF) acids, ketoprofen (KET), dexketoprofen (DKT), diclofenac (DIC), and sulindac (SLD)—and the antibiotic ciprofloxacin (CIP) (Scheme 1) are reported.

There is room for improvement in the pharmacokinetic profiles of all the selected NSAIDs regarding oral administration. MEF and TLF are anthranilic acid derivatives, so-called fenamates. A typical dosage of 500 mg is employed for MEF and it is rapidly absorbed, with a peak plasma concentration attained in 2 to 4 h and elimination half-life of ca. 2 h [7]. On the other hand, TLF works with a typical dosage of 200 mg but shows an oral absorption delay of about 30 min., with a peak plasma concentration attained in 1–2 h and elimination half-life between 8.1 and 13.5 h [8]. KET and DKT are derived from propionic acid. DKT is the S(+) enantiomer of KET, with DKT showing stronger effects [9]. It should be noted that KET is reported to undergo about 10% R to S inversion upon oral administration [10]. These enantiomers also show differences in their pharmacokinetic profiles. KET is rapidly and efficiently absorbed orally, with peak plasma levels attained in 0.5 to 2 h and a half-life of 1 to 3 h, while the maximum concentration of DEX is attained about 30 min after administration and the plasma half-life is about 4–6 h [9,10]. DIC is a phenylacetic acid derivative. Its oral absorption strongly depends on the formulation and food administration, with the peak plasma level occurring between 30 min and several hours [11,12]. It is assumed that only 60% of the drug reaches systemic circulation unchanged, for which the apparent half-life, including all metabolites, is 26–33 h [11]. Finally, SLD is a prodrug that belongs to the arylalkanoic acid class of NSAIDs; it exhibits high and rapid oral absorption, reaching the peak plasma concentration after 1 h of administration and having an elimination half-life between 1.7 and 7 h [13].

CIP is a broad-spectrum antibacterial drug defined as a second-generation fluoroquinolone. Its mechanism of action involves the inhibition of DNA replication through inhibition of bacterial DNA topoisomerase and DNA-gyrase. This relatively new synthetic antibiotic is highly effective against most Gram-negative bacteria, while many Gram-positive bacteria are susceptible or moderately susceptible to it. Moreover, it is often the antibacterial drug of choice when penicillin and macrolide resistance are encountered [14,15]. CIP is classified as a class IV drug in the Biopharmaceutical Classification System, exhibiting low solubility and low permeability [1]. Recent studies attribute the low solubility of CIP to its zwitterionic nature (Scheme 1) and the influence of strong intermolecular H-bonding interactions within the crystal lattice in the solid state [16,17]. Consequently, controlling proton transfer in this kind of zwitterionic drug is essential to modulate solubility. Although it is well-known that the aqueous solubility of CIP is strongly pH-dependent, salification strategies have proved to be the best approach to successfully enhance the solubility, removing the dipole in zwitterions and decreasing the crystal lattice energy [18,19,20]. Note that, when referring to oral-administration drugs, only those pH values corresponding to the gastrointestinal system are pharmaceutically relevant; i.e., pH 1.2 and 6.8 for gastric and intestinal environments, respectively.

As mentioned above, NSAIDs and ciprofloxacin are widely prescribed in clinics. Although the combination of NSAIDs and CIP is generally regarded as safe, it should be noted that there have been several reports suggesting an increased risk of CIP side effects affecting the central nervous system when CIP is administered along with certain NSAIDs, such as ibuprofen. Therefore, patients with renal disease, history of seizures, or other neurological disorders who have been prescribed this combination must be monitored by a physician [21].

The aim of this study was to explore new therapeutic options that would allow the administration of NSAID–CIP combinations with better performance and lower health risks. For this purpose, the solubility of NSAIDs and the efficacy of CIP in novel CIP–NSAID salts were assessed. Moreover, special attention was paid to the structure–property relationships, seeking to rationalize the physicochemical behavior—and, therefore, the pharmacokinetics—of these novel multicomponent pharmaceutical solids through a comprehensive analysis of their crystal structure.

## 2. Results and Discussion

### 2.1. Design of Molecular Salts

A Cambridge Structural Database (CSD version 5.43, update 4 from November 2022) survey based on CIP resulted in 67 hits. After excluding entries corresponding to CIP hydrates, solvates, and polymorphs, as well as CIP metal complexes, the remaining dataset contained 35 molecular salts (52%). The high number of observed molecular salts agrees with the amphoteric nature of CIP, which has basic piperazinyl nitrogen and carboxylic acid groups. In this dataset, the p*K*_a_ of the CIP carboxylic group is less acidic than the H-donor group of the coformers/counterions (mainly carboxylic groups), thus allowing it to remain protonated and form an intramolecular H-bond, promoting proton transfer to the piperazinyl nitrogen and forming a molecular salt. Only two salt structures containing CIP and a NSAID as counterion have been reported: CIP–indoprofen monohydrate and CIP–diflunisal salts [18]. The obtainment of CIP–NSAID salts was expected considering the so-called “p*K*_a_ rule” [5]. This rule, based on experimental crystallographic data, states that salt formation usually occurs when the Δp*K*_a_ between ionizable groups (acid–base) is greater than three p*K*_a_ units, with systems showing a Δp*K*_a_ > 4 usually exclusively forming salts. Although proton transfer can be considered a continuum [22], the application of the p*K*_a_ rule is widely considered in the design of multicomponent pharmaceutical solids [23,24,25]. In Table 1, the reported and calculated p*K*_a_ values for CIP and all the NSAIDs included in this work are detailed. The calculated p*K*_a_ values were rather similar to the experimental ones, showing a difference of at least three p*K*_a_ units between the NSAIDs and the piperazine moiety of CIP. Thereby, the obtained results are consistent with salt formation and in line with the results for other pharmaceutical salts reported for CIP [18,19,20,26].

To evaluate the propensity of CIP to form multicomponent solids with our selection of NSAIDs, a COSMOQuick virtual screening was conducted. This tool is based on thermodynamical calculations that determine the excess enthalpy of mixing or formation (H_ex_) for both APIs in comparison to the pure components in a supercooled liquid phase [39]. Those compounds with negative H_ex_ values are likely to form multicomponent solids. This approach has proved to be a useful tool in the search for new molecular complexes [40,41,42,43]. The results obtained with COSMOQuick (Table 2) confirmed the preference of our NSAID candidates (including those already reported in the literature) to form new compounds with CIP.

### 2.2. Salt Synthesis

Liquid-assisted grinding (LAG) is a widely used mechanochemistry technique that has demonstrated an incredible capacity to synthesize new multicomponent materials quickly and efficiently, with low time requirements and solvent consumption [44]. In this work, a 1:1 stoichiometric mixture of CIP and the corresponding NSAID, along with 50 µL of methanol as liquid additive, was placed in a stainless-steel jar with two stainless-steel balls with 7 mm diameters. After 30 min of LAG, the products were characterized using powder X-ray diffraction (PXRD) and the patterns were compared to those of the parent components in order to determine whether a new material, hereafter referred to as a new phase, had been formed.

**Figure 1:**
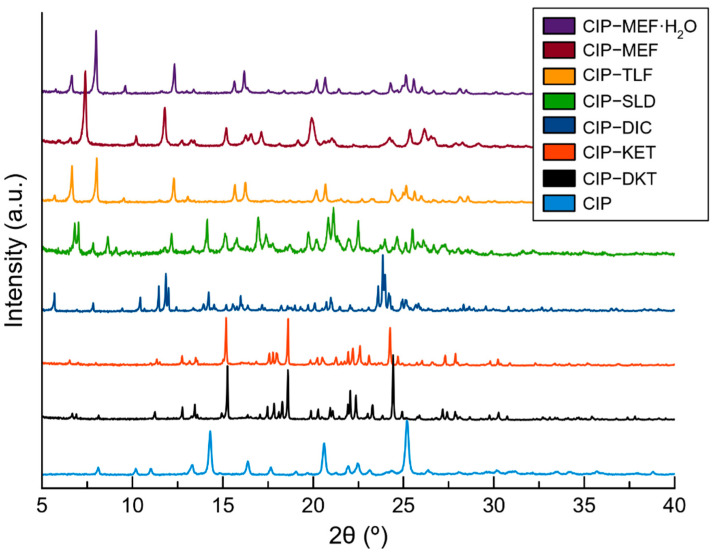
Powder X-ray diffraction (PXRD) patterns for CIP and the new solid forms reported in this work.

Figure 1 shows the PXRD patterns of the materials resulting from the grinding experiments. The comparison showed characteristic peaks that differed from those of the two parent APIs (Figure S1), therefore indicating the formation of new phases. It should be noted that the product of the 1:1 mixture of CIP and DIC resulted in the formation of a new phase with excess CIP, suggesting the formation of a new compound with different stoichiometry from the one initially proposed. LAG of CIP with DIC was repeated with a 1:2 stoichiometric mixture, resulting in a pure phase with no additional peak compared to the parent components.

All the obtained polycrystalline materials were used for recrystallization to obtain single crystals for further structure determination with single-crystal X-ray diffraction (SCXRD). Single crystals were grown via solvent evaporation at room temperature from saturated solutions. Unfortunately, recrystallization of CIP–SLD resulted in the crystallization of the parent components. Different conditions and solvents were used in order to grow a CIP–SLD phase without success. At this point, hydrothermal crystallization was carried out to obtain CIP–SLD single crystals. Previous studies have reported that this technique is a powerful tool that can be used to obtain multicomponent materials through the modification of temperature and pressure parameters [45]. As a result, CIP–SLD crystals of good size and quality were found in the matrix of the aqueous solution and used for SCXRD experiments.

Further crystal-structure determination proved that the product first obtained from the LAG of an equimolar mixture of CIP and MEF corresponded to a monohydrated form of CIP–MEF. After heating this material in an oven at 120 °C for 2 days, a totally different PXRD pattern was observed, suggesting the dehydration of the hydrated salt. This was confirmed by subsequent structure determination using a suitable single crystal of the anhydrated form grown from a saturated acetone solution (Figure S2).

Simulated powder patterns were obtained for all the crystal phases from the corresponding crystal structures and compared with the experimental PXRD patterns. Good agreement between the experimental and the simulated powder patterns was observed, as shown in Figure S1, which confirmed the phase purity of the bulk product obtained from LAG.

### 2.3. Structural Studies of Molecular Salts

Single crystals of molecular salts suitable for structural analysis were obtained, and their structures were determined using SCXRD. The crystallographic data for these salts are summarized in Table S1. Asymmetric units of the salts are depicted in Figures S3–S5, and hydrogen bond information is presented in Table S2. SCXRD data confirmed the proton transfer from the acid groups of the NSAIDs to the piperazine ring of CIP. This was also evidenced in the experimental electron density map and confirmed by the analysis of the C–O bond distances for the carboxylate group in the NSAIDs, with the ΔDC–O values being similar to those observed in salts (typically lower than 0.03 Å) [22].

All the structures showed the expected R_2_NH_2_^+^⋯^−^OOC synthon, with the protonated piperazine nitrogen forming a single or a bifurcated H-bond with the deprotonated carboxylate oxygen of the NSAID molecule. Thus, the formation of supramolecular tetrameric units across inversion centers, described with the graph set motif $R_{4}^{4}\left(12\right)$ [46,47] (Figures S6–S8), was observed as a general feature in all the salts, with the exception of CIP–SLD.

**Figure 2:**
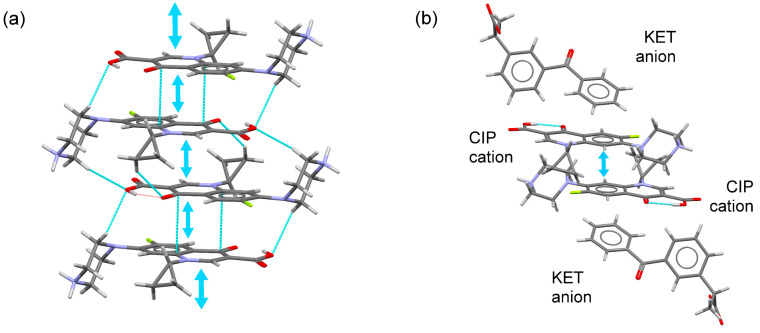
(**a**) Fragment of the infinite CIP column built from π–π stacking interactions. (**b**) A pair of stacked CIP–CIP cations surrounded by NSAID (KET) cations.

The structures of many fluoroquinolone crystals are characterized by arrangements of quinolone molecules in infinite columns that are held together by π–π interactions [26]. This feature was observed in the structures of the salts formed from CIP and MEF, TLF, and DIC (Figure 2a). However, this structural feature was not found for the salts formed from CIP and SLD, KET, and DKT (Figure 2b).

**Figure 3:**
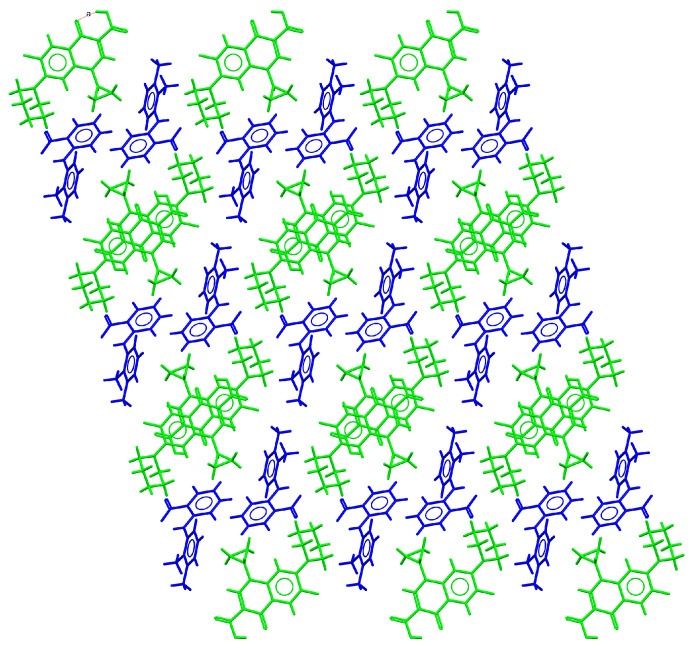
Detailed view of the packing arrangement of CIP cations (green) and MEF anions (blue) in the crystal structure of CIP–MEF.

CIP–MEF crystallized as a molecular salt in the triclinic system spacegroup P-1. The asymmetric unit contained one CIP cation and a mefenamate anion. Two charge-assisted N^+^–H⋯O^−^ hydrogen bonds connected the CIP and MEF ions to form a centrosymmetric tetrameric unit (Figure S6). CIP cations formed infinite columnar structures through head-to-tail π–π stacking interactions between their quinolone moieties. In the crystal structure, the columnar stacks were surrounded by MEF ions and the supramolecular architecture was built through additional C-H··· π interactions between the cyclopropyl ring (CIP) and the substituted aromatic ring of MEF (Figure 3).

**Figure 4:**
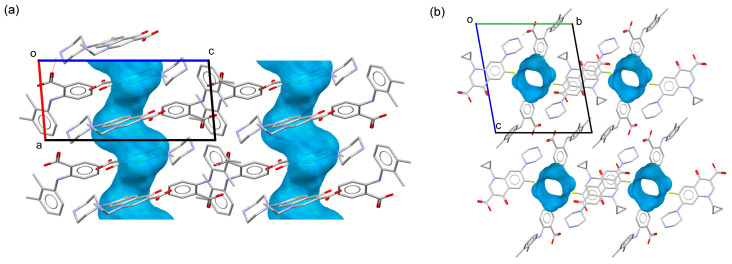
Channels in the crystal structure of CIP–MEF·H_2_O along the b-axis (**a**) and a-axis (**b**) directions mapped with the Voids tool in Mercury [48].

**Figure 5:**
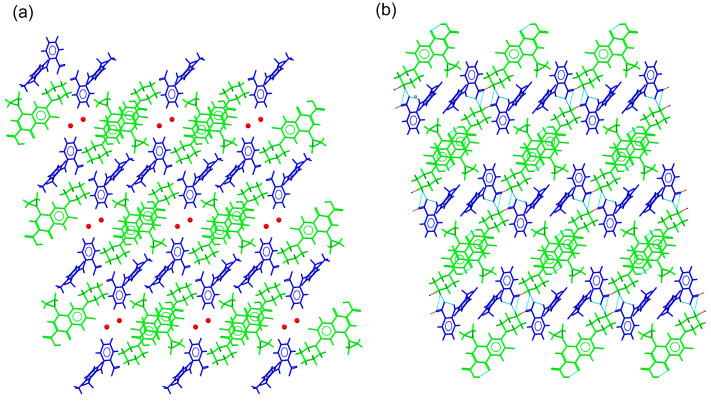
Detailed view of the packing arrangements for (**a**) CIP–MEF·H_2_O and (**b**) CIP–TLF. Green, CIP cations; blue, MEF and TLF anions; red, water molecules.

CIP–MEF·H_2_O also crystallized in the triclinic system spacegroup P-1. The asymmetric unit of this crystal phase contained one CIP cation, a mefenamate anion, and one water molecule, resulting in a salt hydrate closely related to the anhydrous molecular salt previously described. In addition to the formation of tetrameric units and the observed head-to-tail multi-π–π stacking between CIP ions, the crystal structure of this compound exhibited infinite channels extending along the a-axis where the water molecules were hosted (Figure 4), corresponding to 7.7% of the unit cell volume. Similarly to the anhydrous salt, in the 3D crystal structure, the ciprofloxacin columns were surrounded by mefenamic acid molecules, but, in this case, channels containing water molecules (Figure 5) were also present. Likewise, C-H··· π interactions between the cyclopropyl ring of CIP and the substituted aromatic ring of MEF reinforced the supramolecular structure.

CIP–TLF molecular salt crystallized in the triclinic system spacegroup P-1. The asymmetric unit contained one CIP cation and a tolfenamate anion. Both this salt and the salt hydrate with MEF exhibited the same CIP conformation, unlike the related anhydrous CIP–MEF salt. As observed in the CIP–fenamate molecular salts, tetrameric supramolecular units were held together by charge-assisted hydrogen bonds connecting the columns of CIP ions built by π–π stacking interactions (Figure 5). C-H·· π interactions involving the cyclopropyl ring of CIP and the substituted aromatic ring of TLF reinforced this multi-stacked columnar structure.

**Figure 6:**
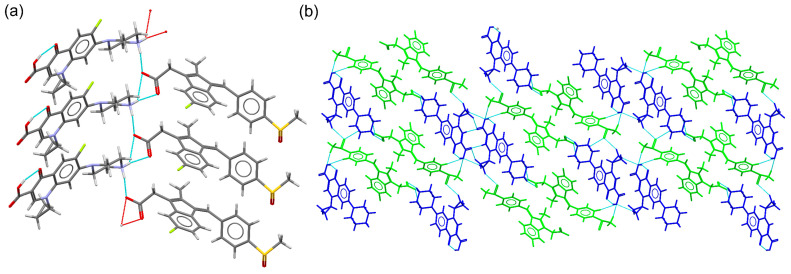
(**a**) Detailed view of the ribbon structure in the molecular salt CIP–SLD. (**b**) Packing arrangement of CIP cations (blue) and SLD anions (green) in the crystal structure of CIP–SLD.

The CIP–SLD molecular salt crystallized in the monoclinic system spacegroup P2_1_/n. The asymmetric unit of CIP–SLD contained one CIP cation and a sulindac anion. Unlike the other structures described in this section, there was formation of tetrameric units or stacked CIP columns. The association between CIP and SLD was maintained by H-bonds that generated a ribbon-like structure, with CIP and SLD ions placed at both ends of the ribbon structure (Figure 6a). These structures were further associated through quinolone environments resulting from centrosymmetric CH···O interactions. Finally, these entities were associated through C-H·· π interactions involving the protonated piperazine ring and the aromatic ring of SLD, generating the 3D supramolecular structure (Figure 6b).

**Figure 7:**
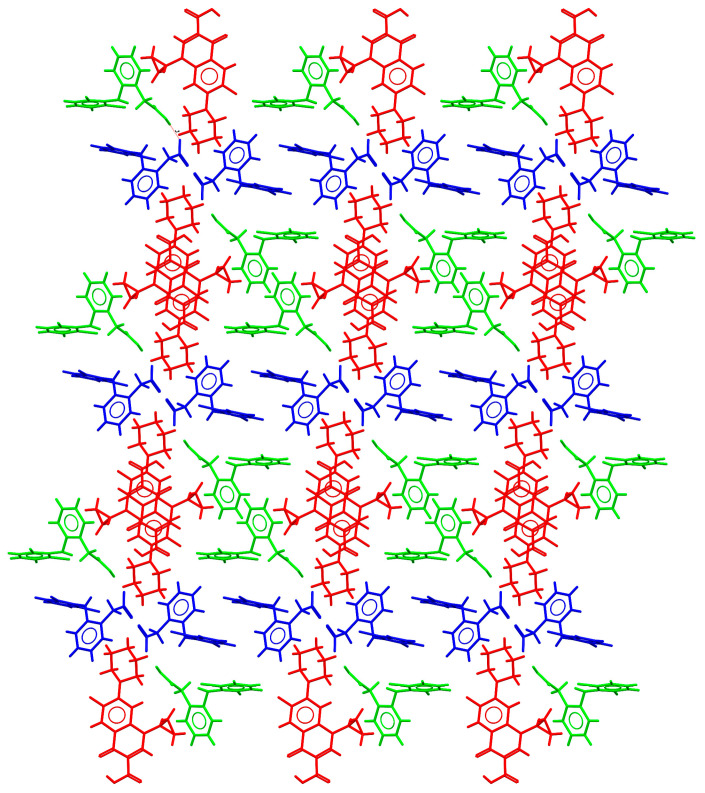
Detailed view of the packing arrangement of CIP cations (red) and DIC anions (blue), forming a grid in the crystal structure of CIP–DIC (neutral DIC molecules in green).

CIP–DIC crystallized in the triclinic system spacegroup P-1 and comprised one CIP cation, a diclofenac anion, and a diclofenac acid neutral molecule in the asymmetric unit. Hence, it can be classified as a conjugate acid/base molecular ionic cocrystal [49]. A tetrameric structure was observed, with additional neutral DIC molecules associated through H-bonding interactions between the DIC acid and DIC anion (Figure S7). This new supramolecular unit was reinforced by C-H·· π and C-H···O(carbonyl) interactions between the cyclopropyl ring of CIP and DIC. As already described for the CIP–fenamate salts, CIP ions associated with each other to generate infinite head-to-tail stacked columns. The resulting supramolecular structure consisted of a grid structure built by the ions, hosting neutral DIC molecules in the voids (Figure 7).

**Figure 8:**
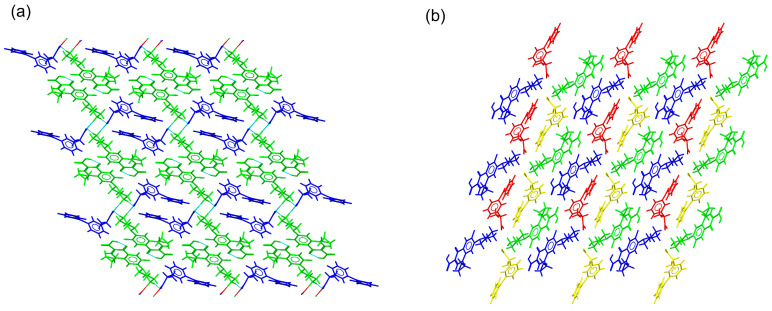
Detailed view of the packing arrangements for (**a**) CIP–KET (CIP cations in green, KET anions in blue) and (**b**) CIP–DKT (CIP cations in green and blue, DKT anions in red and yellow).

The asymmetric unit of CIP–KET contained one CIP cation and a ketoprofen anion. Although tetrameric units were observed, CIP ions were associated in pairs through π–π stacking interactions but did not form infinite columns. KET anions interrupted the formation of the stacked columns, occupying the space between CIP stacked pairs. C-H···O (carbonyl) interactions involving CIP–CIP and KET–KET pairs reinforced the 3D structure (Figure 8a).

CIP–DKT crystallized in the triclinic system spacegroup P1. The asymmetric unit comprised two crystallographically independent pairs of CIP cations and DKT anions. Charge-assisted N^+^–H⋯O^−^ hydrogen bonds connected the CIP and DKT ions to form centrosymmetric tetrameric units. π–π stacking interactions connected the quinolone moieties of the CIP pairs. These pairs were then further surrounded by DKT ions, avoiding the formation of stacked columns (Figure 8b). This feature was in good agreement with the behavior reported for CIP salts with more than one independent molecule in the asymmetric unit [26]. In the crystal structure, additional C-H⋯O(carbonyl) interactions between CIP–CIP and DKT–DKT pairs contributed to the cohesion of the supramolecular structure.

### 2.4. Thermal Stability

**Figure 9:**
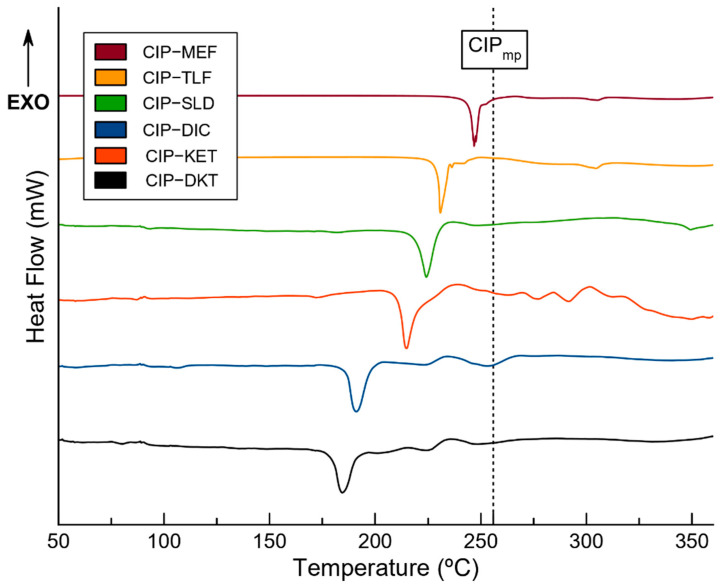
DSC traces for the pure molecular salts and the melting point of the CIP drug [51] (black dotted line).

Thermal stability refers to the capacity of novel phases to maintain their integrity after temperature variations; i.e., below the melting point of the corresponding molecular salts. The thermal behavior and melting point of the new drug–drug salt phases were studied using differential scanning calorimetry (DSC). Figure 9 shows the DSC traces for the new materials. Each DSC trace shows one single endothermic event ascribable to the melting point of the sample, consistent with the phase purity of the powders suggested by the PXRD. In addition, thermogravimetric analyses (TGAs) confirmed the integrity of the molecular salts below the melting point from the absence of weight loss (decomposition) during the period (Figure S8).

Interestingly, a positive correlation between the melting point of the NSAID molecule and the corresponding phase was observed. The melting points of the components decreased in the order: CIP > MEF > TLF > SLD > DIC > KET > DKT (CIP: 256 °C, MEF: 230 °C, TLF: 207 °C, SLD: 183 °C, DIC: 156–158 °C, KET: 94 °C and DKT: 75 °C). The corresponding phases followed the same order, with melting points of 246 °C for CIP–MEF, 230 °C for CIP–TLF, 226 °C for CIP–SLD, 214 °C for CIP–DIC, 191 °C for CIP–KET, and, finally, 184 °C for CIP–DKT.

In all cases, the new solid phases had melting points between those of the two parent APIs. This was particularly noticeable for KET and DKT, for which the melting points increased from 94 and 75 °C to 191 and 184 °C in the combined salts. Similar results have already been described previously, demonstrating that the formation of multicomponent materials has the potential to modulate the melting point and improve the thermal stability of APIs [50].

### 2.5. Thermodynamic Stability

The thermodynamic stability of the novel salts was studied with both slurry in aqueous solution and accelerated ageing condition experiments. These experiments aimed to assess the integrity of the molecular salts when dissolved in aqueous media—i.e., molecular salts in chemical equilibrium—without experimenting with the dissociation of the corresponding APIs or transitioning to other phases. For this purpose, excess amounts of the obtained molecular salts were stirred in deionized water at 25 °C and dissolved until saturation. Figure S9 shows the PXRD patterns for the resulting solid excess, filtered and air-dried, after dissolution (24 h of the slurry experiment). It was observed that CIP–SLD and CIP–MEF·H_2_O demonstrated transitions to thermodynamically more stable species in water. For instance, CIP–SLD turned into an unknown phase, while CIP–MEF·H_2_O transformed into CIP–MEF. This latter finding was in good agreement with the crystal structure of CIP–MEF·H_2_O, which showed a channel hydrate from which H_2_O molecules could easily evacuate upon heating without altering crystallinity, resulting in the anhydrous salt. Furthermore, these results were consistent with the thermogravimetric analysis (TGA) of the salt hydrate, which revealed an initial mass loss below 100 °C (1.3272%) corresponding to the dehydration process, implying the release of 0.43 H_2_O molecules (Figure S10).

Interestingly, the remaining salts (CIP–MEF, CIP–TLF, CIP–DIC, CIP–KET, and CIP–DKT) remained unaltered under the experimental conditions. These observations suggest that salt formation with the selected NSAIDs increased the thermodynamic stability of CIP in aqueous solution.

Experiments under accelerated ageing conditions were performed at 40 °C and 75% relative humidity (RH) for 2 months in order to evaluate possible transformations of the reported molecular salts into other solid phases—i.e., hydrates and polymorphs—upon exposure to the employed RH and temperature. CIP was also included in the experiments to assess the formation of hydrated forms. The PXRD patterns of the samples were periodically recorded and compared with the calculated powder patterns to evaluate their thermodynamic stability (Figure S11). Under these conditions, no changes were observed, thus confirming the integrity of the novel NSAID–CIP solids under ageing conditions.

### 2.6. Solubility Studies

Solubility is a thermodynamic property of solids with significant consequences for the pharmaceutical industry [52], particularly with regard to the oral bioavailability of APIs. Indeed, recently developed API molecules tend to have poor solubility at physiological pH, which is an important factor to consider and optimize. There are several techniques that are currently used to address this issue [53,54,55,56], including the formation of multicomponent materials, such as cocrystals and salts [57]. Unfortunately, the CIP–SLD and CIP–MEF·H_2_O phases were unsuitable for solubility studies since, as already mentioned, CIP–SLD transformed into an unknown phase and CIP–MEF·H_2_O was converted into the anhydrous CIP–MEF salt in aqueous solution. Therefore, only the solubilities of the remaining molecular salts were determined using HPLC analysis, with the results being presented in Table 3. Solubility studies were carried out only at physiological pH values and those pharmaceutically relevant for oral administration; i.e., pH 1.2 and 6.8 for gastric and intestinal environments, respectively. None of the investigated molecular salts underwent a solution-mediated transformation during the study. The congruent solubility of the salts was confirmed by analyzing the solid phase recovered after the experiment with PXRD.

The solubility obtained for CIP at pH 6.8 was consistent with other values reported under the same conditions, thus confirming the validity of the method (Figures S12 and S13, Table S3) [19]. Table 3 shows a comparison of the solubility values of the parent APIs and the novel molecular salts. At pH 6.8, almost all the studied crystal phases exhibited enhanced CIP solubility. The results for the CIP–DKT salt at pH 6.8 should be noted, as the CIP was up to 5.5 times more soluble. In contrast, the CIP–TLF and CIP–DIC salts did not show solubility enhancements for CIP, and there was no apparent relationship between the solubility sets and the corresponding NSAID structures. On the other hand, solubility enhancement was evident when looking at the solubility of the NSAIDs, with the CIP–DIC salt being up to 20.8 times more soluble than the reference API at pH 6.8.

As expected, due to its zwitterionic nature, the solubility of CIP increased significantly at pH 1.2 [68]. Likewise, enhanced solubility was observed for CIP in all the molecular salts under the studied conditions. Unfortunately, solubility values for the isolated NSAIDs could not be obtained at pH 1.2 due to extreme insolubility. Therefore, a qualitative comparison of the solubility enhancements of the NSAIDs at pH 1.2, as presented in Table 3 for pH 6.8, could not be undertaken. Nonetheless, the fact that all novel CIP–NSAID molecular salts maintained their integrity at pH 1.2 qualitatively confirmed the solubility enhancement of the NSAIDs when formulated as multicomponent pharmaceutical solids. However, absorption of NSAIDs mainly takes place in the small intestine [6]; hence, the results at pH 6.8 are more relevant for pharmaceutical purposes. The final pH levels of the solutions after the solubility experiments were measured and revealed no significant changes (Table S6).

There is a correlation between the solubility of the molecular salts and that of the corresponding NSAIDs. Moreover, the solubility of the salts was inversely related to their melting points; i.e., the solubility increased as the melting point decreased. This latter finding highlights that solubility enhancement might be ascribable to the crystal lattice energy of the reported salts. Such behavior has been reported previously [19,69]. The high solubility of the studied salts can be attributed to the salification effect. The presence of charge-assisted hydrogen bonds and the supramolecular arrangement in the crystal structure effectively disrupted the formation of strong API homosynthons, which was responsible for the low aqueous solubility observed in the model parent drugs.

### 2.7. Antimicrobial Activity

**Figure 10:**
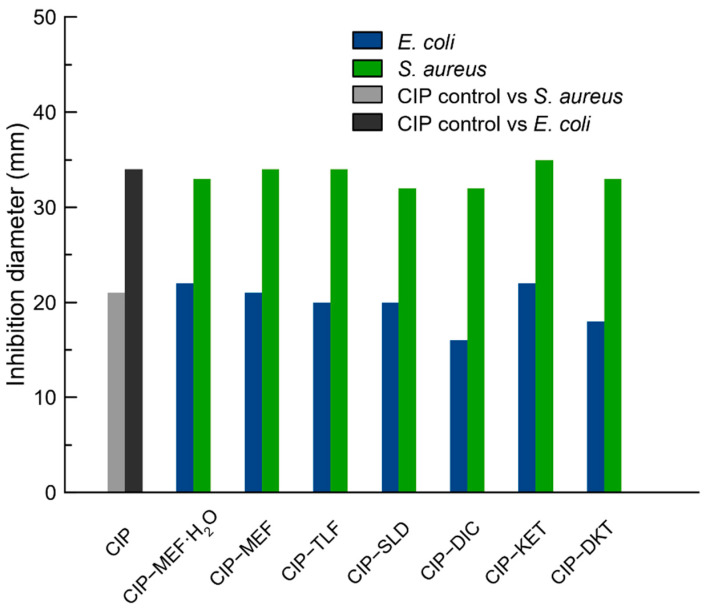
Antibiogram of CIP and the reported molecular salts tested against *S. Aureus* and *E. Coli*.

The antimicrobial activity of the molecular salts reported in this work was evaluated and compared to that of CIP using microorganisms with medical relevance: *Staphylococcus aureus* (ATCC 9144) and *Escherichia coli* (CECT 101). Figure 10 summarizes the results of this experiment, in which CIP, as expected, demonstrated noticeably higher antimicrobial activity against Gram-negative bacteria (*E. coli*) than against Gram-positive bacteria (*S. aureus*). However, it was able to efficiently inhibit the microbial proliferation of both types of bacteria.

The molecular salts presented in this work showed similar antibacterial activity as CIP alone (Figure 10, Table S4), which was also evidenced by the inhibition halos obtained on TSA agar cultures, as shown in Figure S14. However, while CIP alone required 2.5 µg to exert such antimicrobial inhibition, the CIP concentration in the CIP–NSAIDs molecular salts was proportionally reduced. For instance, the CIP–DKT salt was capable of exhibiting the same inhibitory effect as CIP alone but using only 1.41 µg of CIP (Table S5). The contribution of CIP in each CIP–NSAID salt was calculated based on the molecular weight and given in Table S5. In this context, the case of CIP–DIC should be noted: the CIP contribution was reduced to 0.90 µg due to the 1:2 stoichiometry of this phase, which contained approximately three times less CIP than the control.

These results confirmed that salt formation with NSAIDs not only keeps the antibacterial activity of CIP intact but also reduces the amount of CIP needed to achieve the same inhibitory effect as a result of the improvement in the salts’ solubility, which also enhanced their diffusion in the culture.

## 3. Materials and Methods

### 3.1. Materials

All the APIs used in this work were obtained from a commercial TCI-Europe source and used as received. All solvents were purchased from Sigma-Aldrich and were also used as received.

Synthesis of diclofenac acid form: the diclofenac acid (DIC) form was obtained through the hydrolysis of sodium diclofenac (DICNa), dissolving 5 mol of DICNa (1.590 g) in 30 mL of ultrapure water (Milli-Q, Millipore, Burlington, MA, USA) at 40 °C. HCl was added dropwise at 1 M to the solution until no more diclofenac precipitated. The polycrystalline powder was filtrated and washed with cold deionized water and then allowed to dry at 35 °C for 24 h. Powder X-ray diffraction (PXRD) was used to confirm the purity of DIC.

### 3.2. Mechanochemical Syntheses

Mechanochemical synthesis was conducted via liquid-assisted grinding (LAG) in a Retsh MM2000 ball mill operating at 25 Hz frequency, using stainless-steel jars along with stainless-steel balls with diameters of 7 mm. All syntheses were repeated to ensure reproducibility.

Synthesis of CIP–MEF·H_2_O, CIP–TLF, CIP–SLD, CIP–KET, and CIP–DKT salts: mixtures of CIP (0.2 mmol, 66.27 mg) and the corresponding NSAID were placed in stainless-steel jars along with two steel balls with diameters of 7 mm and 50 µL of methanol (1:1 stoichiometric ratio). The mixtures were then milled for 30 min.

Synthesis of CIP–MEF salt: a mixture of CIP (0.2 mmol, 66.27 mg) and MEF (0.2 mmol, 48.26 mg) was placed in a stainless-steel jar along with two steel balls with diameters of 7 mm and 50 µL of methanol (1:1 stoichiometric ratio). After 30 min of milling, the product was collected and heated at 120 °C for 2 days.

Synthesis of CIP–DIC cocrystal salt: a mixture of CIP (0.2 mmol, 66.27 mg) and DIC (0.4 mmol, 118.46 mg) was placed in a stainless-steel jar along with two steel balls with diameters of 7 mm and 50 µL of methanol (1:2 stoichiometric ratio). The mixture was then milled for 30 min.

Bulk materials were further evaluated using PXRD to determine whether new materials had been formed.

### 3.3. Preparation of Single Crystals

Single crystals of CIP–MEF_H2O_ and CIP–TLF were obtained by dissolving 0.5 mmol (16.56 mg) and 0.5 mmol of MEF and TLF, respectively (12.06 and 13.08 mg), in a 1:1 (*v*/*v*) mix of acetonitrile/methanol. Crystals suitable for single-crystal X-ray diffraction (SCXRD) appeared after 2 days of slow solvent evaporation at room temperature.

Single crystals of CIP–MEF were obtained from a saturated solution of the product of the dehydration of CIP–MEF·H_2_O in acetone. Crystals suitable for SCXRD appeared after 2 days of slow solvent evaporation at room temperature.

Single crystals of CIP–KET and CIP–DIC were obtained from a saturated solution of the product of the LAG in methanol. Crystals suitable for SCXRD appeared after 1 day of slow solvent evaporation at room temperature.

Single crystals of CIP–SLD were obtained through hydrothermal synthesis. A mixture of CIP (0.1 mmol, 33.10 mg) and SLD (0.1 mmol, 35.64 mg) (1:1 stoichiometric ratio), was placed in a hydrothermal reactor with 3 mL of distilled water. The reactor was tightly sealed and heated at 110 °C for 24 h. The reactor was then cooled down to room temperature before opening. Crystals of CIP–SLD suitable for single-crystal X-ray diffraction were directly obtained from the hydrothermal reactor.

### 3.4. Powder X-ray Diffraction (PXRD)

PXRD experiments were performed for the samples with a Bruker D8 Advance Vαrio diffractometer (Bruker-AXS, Karlsruhe, Germany) equipped with a LYNXEYE detector and Cu-K_α1_ radiation (1.5406 Å). Diffraction patterns were collected over a 2θ range of 5–40° using a continuous step size of 0.02° and a total acquisition time of 30 min.

### 3.5. Single-Crystal X-ray Diffraction (SCXRD)

Measured crystals were prepared under inert conditions and immersed in perfluoropolyether as the protecting oil for manipulation. Suitable crystals were mounted on MiTeGen Micromounts™ and these samples were used for data collection. Data were collected with a Bruker D8 Venture diffractometer. The data were processed with the APEX4 suite [70]. The structures were solved using intrinsic phasing [71], which revealed the position of all non-hydrogen atoms. These atoms were refined on F^2^ with a full-matrix least-squares procedure using anisotropic displacement parameters [72]. All hydrogen atoms were located in difference Fourier maps and included as fixed contributions riding on attached atoms with isotropic thermal displacement parameters 1.2 or 1.5 times those of the respective atom. Olex2 software was used as a graphical interface [73]. Geometric calculations and production of molecular graphics were undertaken with Mercury [48] and Olex2 [73]. The crystallographic data for the reported structures were deposited with the Cambridge Crystallographic Data Center as supplementary publication no. CCDC 2232207-2232213. Additional crystal data are shown in Table S1. Copies of the data can be obtained free of charge at https://www.ccdc.cam.ac.uk/structures/, accessed on 22 December 2022.

### 3.6. Differential Scanning Calorimetry and Thermogravimetric Analysis

Differential scanning calorimetry (DSC) and thermogravimetric analysis (TGA) were performed with a Mettler-Toledo TGA/DSC 3+ Star analyzer (Mettler Toledo, Columbus, OH, USA). Experimental conditions: aluminum crucibles of 40 µL volume, dry nitrogen atmosphere with 50 mL/min flow rate, heating rate of 10 °C/min. The calorimeter was calibrated with 99.99% purity indium (m.p.: 156.4 °C; DH: 28.14 J/g).

### 3.7. Slurry and Ageing Condition Experiments

Thermodynamic stability in aqueous solution was evaluated through slurry experiments. Excess powder samples of each phase were added to 1 mL of water and stirred for 24 h in sealed vials. The solids were collected, filtered, dried, and analyzed with PXRD.

Likewise, thermodynamic stability was evaluated under accelerated ageing conditions: 200 mg of solid was placed in watch glasses and left at 40 °C in 75% relative humidity in a Memmert HPP110 climate chamber (Memmert, Schwabach, Germany). The integrity of the solid forms under the above accelerated ageing conditions was periodically monitored using PXRD for two months.

### 3.8. Solubility Studies

A saturated solution of each molecular salt was prepared by adding the solid excess of each phase to water solutions at pH 1.2 (hydrochloric buffer) and pH 6.8 (PBS buffer) and stirring for 24 h at 25 °C in a water bath until thermodynamic equilibrium was reached. After that, the solution was filtered through 0.45 μm syringe filters and directly used for the chromatographic analysis to determine the CIP concentration under equilibrium conditions. Drug concentration was analyzed using high-performance liquid chromatography (HPLC). The mobile phase consisted of phase A (MilliQ water with 0.1% formic acid) and phase B (acetonitrile) and was used in an isocratic mode with an 80:20 ratio. The details of the experimental conditions are summarized in the Supplementary Materials in Table S3.

### 3.9. Evaluation of Antimicrobial Activity

The antimicrobial susceptibility of the different salts was assayed against *Staphylococcus* aureus (ATCC 9144) and *Escherichia coli* (CECT 101) using the Kirby–Bauer diffusion agar method [74]. The strains were cultured in TSA agar and two to three colonies of each were suspended in NaCl 0.9% solution, which was adjusted by comparing it with a 0.5 McFarland standard (1.5 × 108 bacteria/mL), and then plated on Mueller–Hinton agar plates. Sterile filter papers with a diameter of 5 mm were respectively impregnated with 2.5 µL of a DMSO solution of each salt with a final concentration of 1 mg/mL, resulting in a final solid amount of 2.5 µg for each solid. A sterile filter paper was impregnated with 2.5 µL of DMSO as a control. The plates were incubated overnight at 37 °C, and the clear zones around the filter papers were measured in millimeters.

## 4. Conclusions

The combination of mechanochemical synthesis and salification was confirmed to be an optimal drug synthesis approach to obtain enhanced multicomponent pharmaceutical materials. The novel NSAID–CIP solids showed not only improved solubility and thermal stability compared to the parent APIs but also achieved better efficiency, keeping the same antibiotic efficacy with reduced dosages.

When comparing all the novel NSAID–CIP solids, CIP–SLD and CIP–MEF turned out to be the less promising materials due their conversion into other products in aqueous solution. The weaknesses of these solids were rationalized according to their intimate crystal structure; for instance, CIP–SLD presented a more exposed ribbon-like structure, while the CIP–MEF salt hydrate structure was extremely close to the anhydrous molecular salt.

However, the results for the CIP–DKT and CIP–DIC molecular salts were quite remarkable, showing particularly high solubility (5.5 times higher than CIP for CIP–DKT and 20.8 times higher than DIC for CIP–DIC) along with good thermodynamic stability in solution and under ageing conditions. The enhanced solubility could also be rationalized in terms of the structure–property relationship. The charge-assisted H-bonds within the supramolecular arrangement effectively promoted the formation of new heterosynthons that tailored the crystal lattice energy. It should be noted that enhanced solubility means the administration dose can be lowered, which is considered a great advantage in terms of potential reductions in the dose-dependent side effects usually linked to NSAID oral administration.

These promising results open the door to further development of the selected drug–drug multicomponent candidates as potential fixed-dose drug combinations.

## Figures and Tables

**Table 1:** Molecules used to make salts with CIP and their corresponding p*K*_a_ values.

API	Reported p*K*_a_ Values	Ref.	Calculated p*K*_a_ Values *	Δp*K*_a_
Ciprofloxacin	8.74 (−NH)	[27]	8.77	--
Dexketoprofen	3.88; 4.55; 5.02	[28,29,30]	3.88	4.86
Mefenamic acid	3.93; 4.64	[31]	3.89	4.81
Tolfenamic acid	4.3; 5.11	[32,33]	3.88	4.44
Ketoprofen	3.8; 3.7; 4; 4.30	[31,33,34,35]	3.88	4.94
Diclofenac	4.15; 4.2	[33,36]	4.00	4.59
Sulindac	4.7	[30,37]	4.09	4.04

* Estimated using the p*K*_a_ plugin in MarvinSketch software [38].

**Table 2:** Ranking positions for CIP coformer candidates, including the two NSAID drugs reported in CSD (in bold), based on COSMOQuick calculations.

Coformer	H_ex (kcal/mol)	Reference
**Diflunisal**	−3.80753	[17]
Tolfenamic acid	−2.718395	This work
Diclofenac	−3.069495	This work
Mefenamic acid	−2.540215	This work
**Indoprofen**	−1.520345	[17]
Ketoprofen	−1.530325	This work
Dexketoprofen	−1.530325	This work
Sulindac	−0.260615	This work

**Table 3:** Solubility of the molecular salts determined with HPLC analysis.

Compound	Experimental (HPLC) CIP Solubility pH 6.8 (mg/mL)	Experimental (HPLC) CIP Solubility pH 1.2 (mg/mL)	Solubility Enhancement Compared to CIP/NSAIDs (pH 6.8)	Reported NSAID Solubility (mg/mL)	Ref.
CIP–DKT	0.465	9.980	5.5x/-	Not reported	
CIP–KET	0.207	9.533	2.4x/0.8x	<0.2	[58,59,60,61,62]
CIP–MEF	0.140	1.182	1.7x/2x	<0.05	[63,64]
CIP–TLF	0.047	6.567	0.6x/0.74x	<0.05	[63]
CIP–DIC	0.035	5.946	0.4x/20.8x	<0.003	[60,61,65,66]
CIP	0.084	14.671		0.08	[67]

References apply to solubility values reported in the literature for the studied NSAIDs alone, which are given in the previous column.

## Data Availability

The crystallographic information file with the structural data for the new phase can be obtained from the CCDC and requested with the references 2232207-2232213 at https://www.ccdc.cam.ac.uk/structures/, accessed on 22 December 2022.

## Supplementary Materials

The following are available online at https://www.mdpi.com/article/10.3390/ijms24043305/s1.
