# Supplementary material for: Enhanced NSAIDs Solubility in Drug–Drug Formulations with Ciprofloxacin
Source: Int J Mol Sci. 2023 Feb 7;24(4):3305. doi: 10.3390/ijms24043305 (PMC9964002; doi:10.3390/ijms24043305)
Supplement: Supplementary file 1 [file ijms-24-03305-s001.zip › ijms-2150094-supplementary.pdf]

International Journal of
Molecular Sciences

MDPI

Article

Enhanced NSAIDs solubility in drug-drug formulations with ciprofloxacin

Francisco Javier Acebedo-Martínez [1], Alicia Domínguez-Martín [2], Carolina Alarcón-Payer [3], Alejandro Sevillano-Páez [1,2], Cristóbal Verdugo-Escamilla [1], Josefa María González-Pérez [2], Fernando Martínez-Checa [4,5,*], Duane Choquesillo-Lazarte [1,*]

Table of contents

Figure S1. PXRD patterns of the molecular salts obtained by LAG, compared with their respective components.

Figure S2. PXRD patterns of the salt hydrate CIP–MEF·H_2O obtained by liquid-assisted grinding (LAG), the simulated pattern from crystal structure and the related salt CIP–MEF.

Figure S3. ORTEP representation showing the asymmetric unit of CIP—MEF (left), CIP—MEF·H_2O (center) and CIP—TLF (right) with atom numbering scheme (thermal ellipsoids are plotted with the 50% probability level).

Figure S4. ORTEP representation showing the asymmetric unit of CIP—SLD (left) and CIP—DIC (right) with atom numbering scheme (thermal ellipsoids are plotted with the 50% probability level).

Figure S5. ORTEP representation showing the asymmetric unit of CIP—KET (left) and CIP—DKT (right) with atom numbering scheme (thermal ellipsoids are plotted with the 50% probability level).

Figure S6. Tetrameric supramolecular unit in the crystal structures of CIP—MEF (left), CIP—MEF·H2O (center) and CIP—TLF (right).

Figure S7. Tetrameric supramolecular unit in the crystal structures of CIP—DIC (top left), CIP—KET (top right) and CIP—DKT (bottom).

Figure S8. DSC and TG traces of the reported molecular salts.

Figure S9. PXRD patterns of the reported molecular salts after aqueous slurrying for 24 hours.

Figure S10. TG analysis of the hydrate salt CIP—MEF·H_2O.

Figure S11. PXRD patterns of reported molecular salts under accelerated ageing conditions for 2 months.

Figure S12. Chromatogram of CIP—DKT salt showing a first peak corresponding to CIP and a second peak corresponding to DKT.

Figure S13. Calibration curve of CIP determined from HPLC data.

Figure S14 Antimicrobial activity of CIP and CIP phases on TSA agar plates cultured with *Staphylococcus* aureus (ATCC 9144, first row) and *Escherichia coli* (CECT 101, second row).

Table S1. Crystallographic data and structure refinement details of CIP—NSAIDs molecular salts.

Table S2. Hydrogen bonds for CIP—NSAIDs molecular salts [Å and deg.].

Table S3. HPLC method parameters.

Experimental procedure for determination of solubility by HPLC analysis.

Table S4. Zone of inhibition values of CIP and the reported molecular salts.

Table S5. Contribution of CIP in each CIP–NSAID molecular salt.

Table S6. Final pH values measured during solubility determination by HPLC analysis.

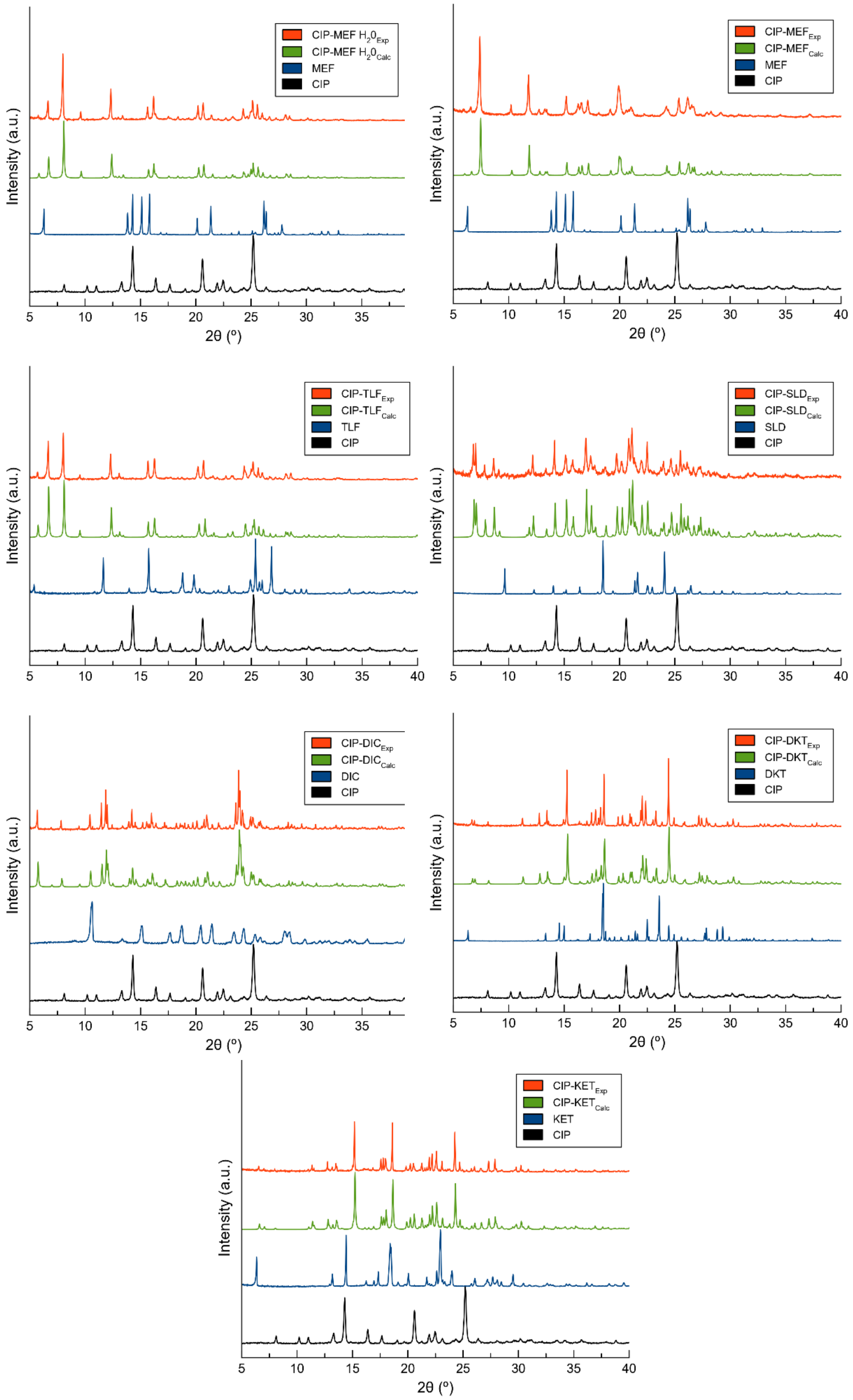

Figure S1. PXRD patterns of the molecular salts obtained by LAG, compared with their respective components.

International Journal of
Molecular Sciences

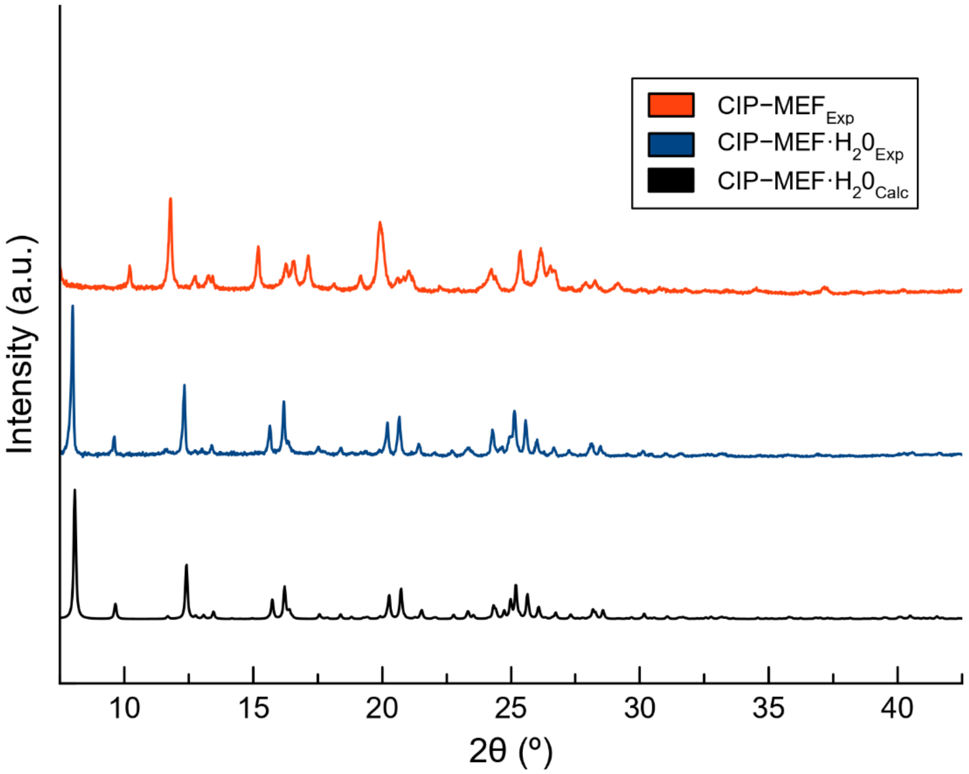

Figure S2. PXRD patterns of the salt hydrate CIP–MEF·H_2O obtained by liquid-assisted grinding (LAG), the simulated pattern from crystal structure and the related salt CIP–MEF.

International Journal of *Molecular Sciences*

MDPI

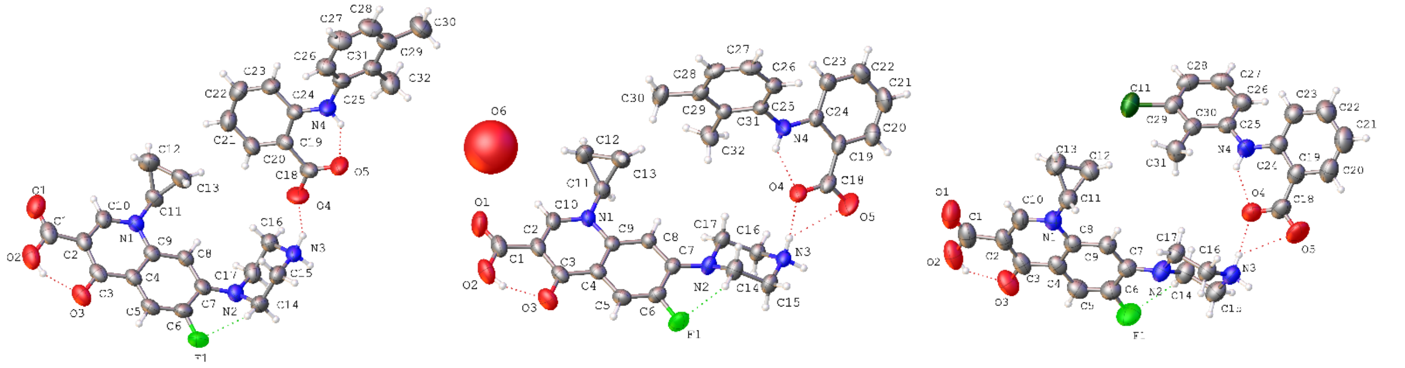

Figure S3. ORTEP representation showing the asymmetric unit of CIP—MEF (left), CIP—MEF·H_2O (center) and CIP—TLF (right) with atom numbering scheme (thermal ellipsoids are plotted with the 50% probability level).

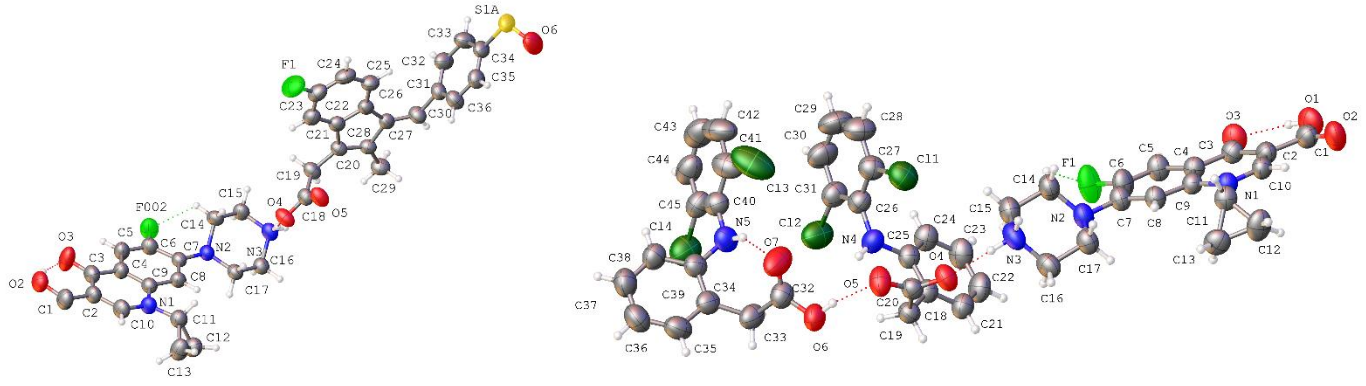

Figure S4. ORTEP representation showing the asymmetric unit of CIP—SLD (left) and CIP—DIC (right) with atom numbering scheme (thermal ellipsoids are plotted with the 50% probability level).

International Journal of *Molecular Sciences*

MDPI

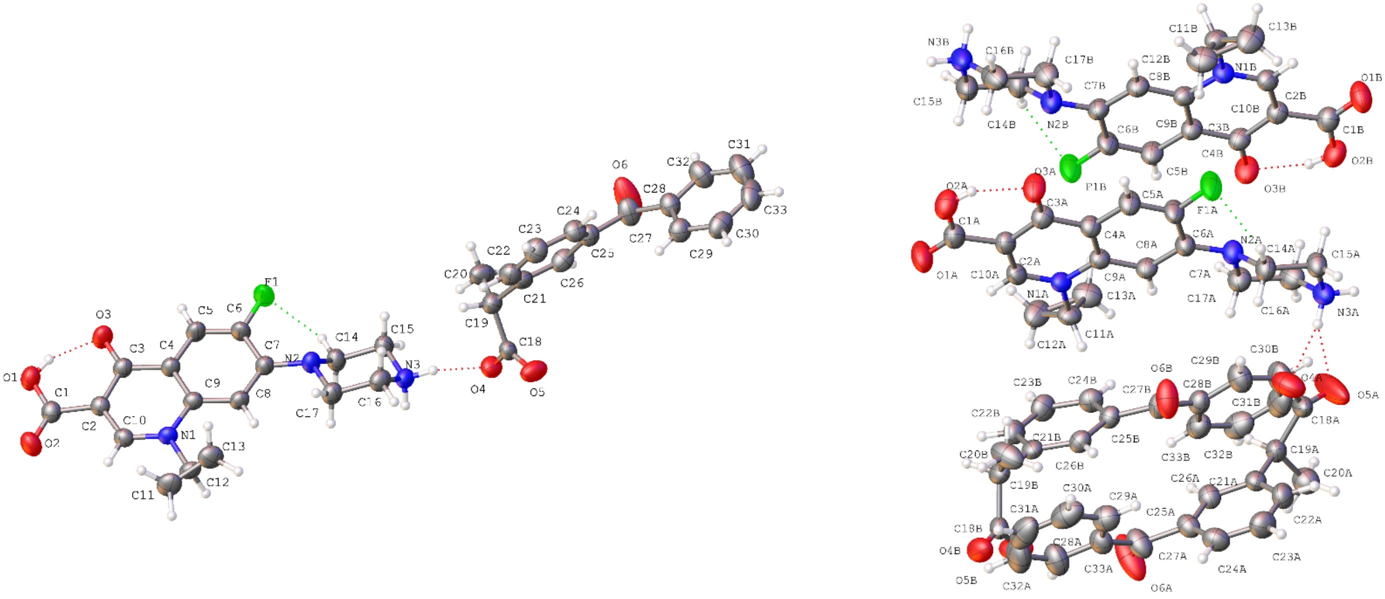

Figure S5. ORTEP representation showing the asymmetric unit of CIP—KET (left) and CIP—DKT (right) with atom numbering scheme (thermal ellipsoids are plotted with the 50% probability level).

International Journal of
Molecular Sciences

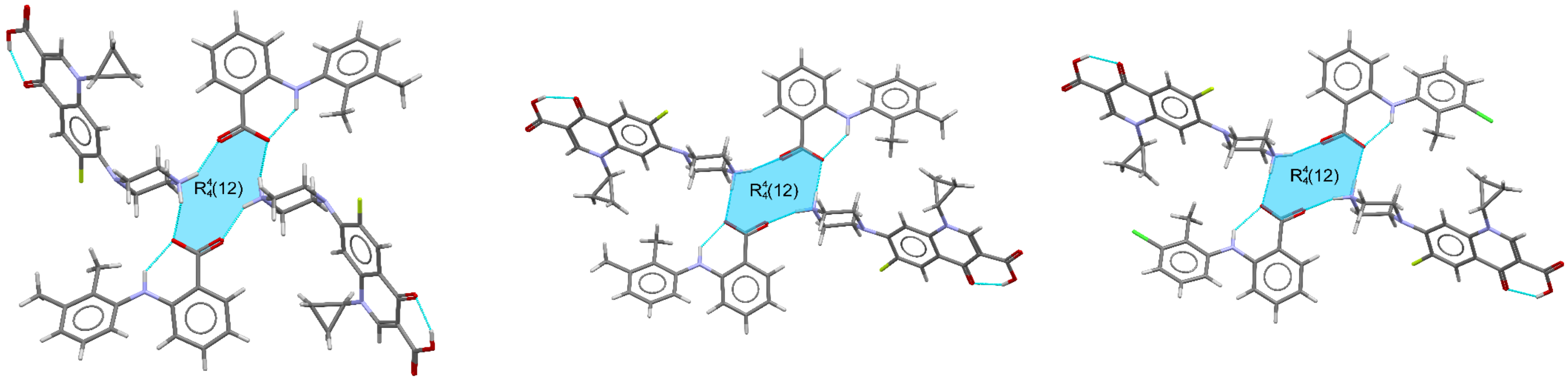

Figure S6. Tetrameric supramolecular unit in the crystal structures of CIP—MEF (left), CIP—MEF·H2O (center) and CIP—TLF (right).

International Journal of
Molecular Sciences

MDPI

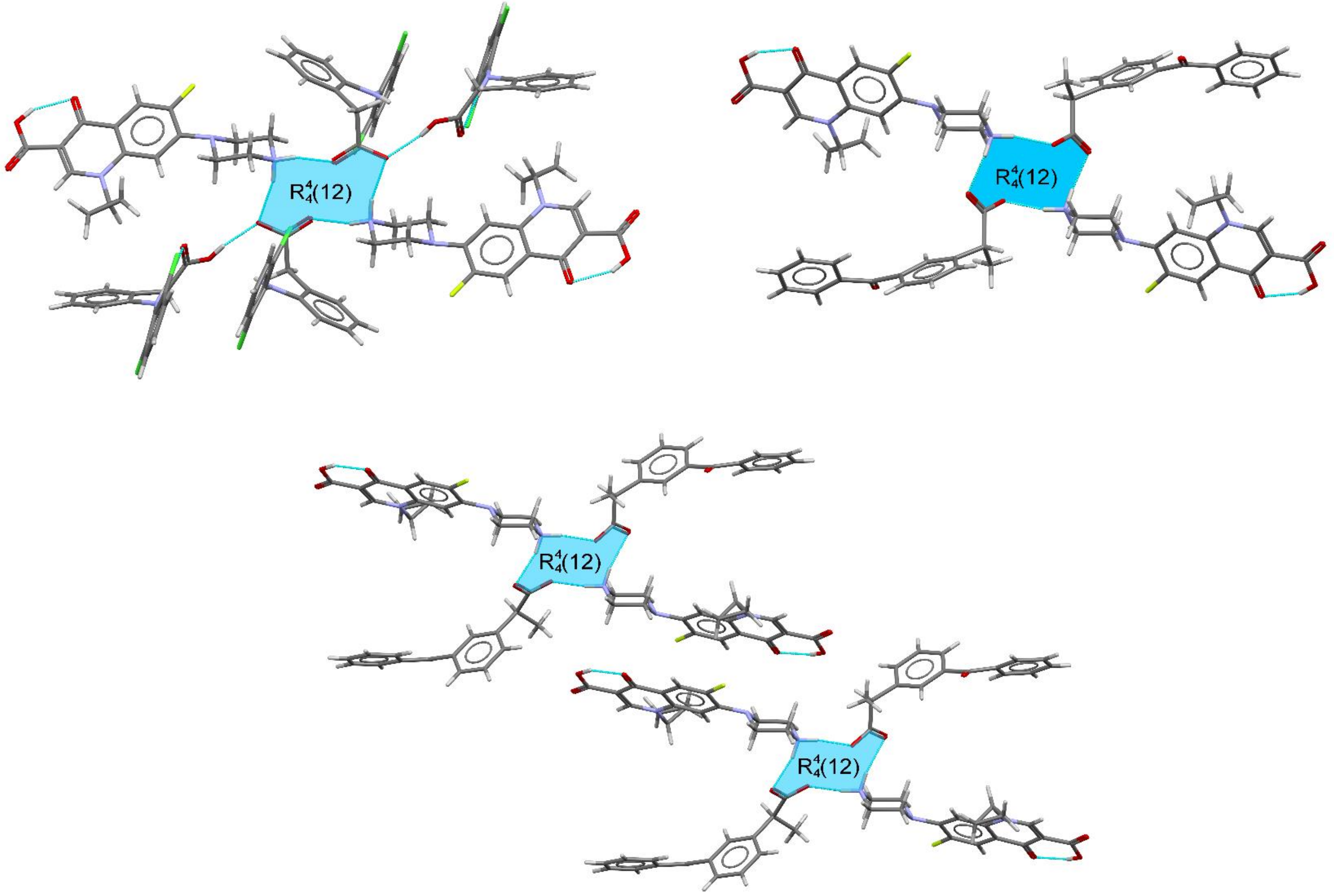

Figure S7. Tetrameric supramolecular unit in the crystal structures of CIP—DIC (top left), CIP—KET (top right) and CIP—DKT (bottom).

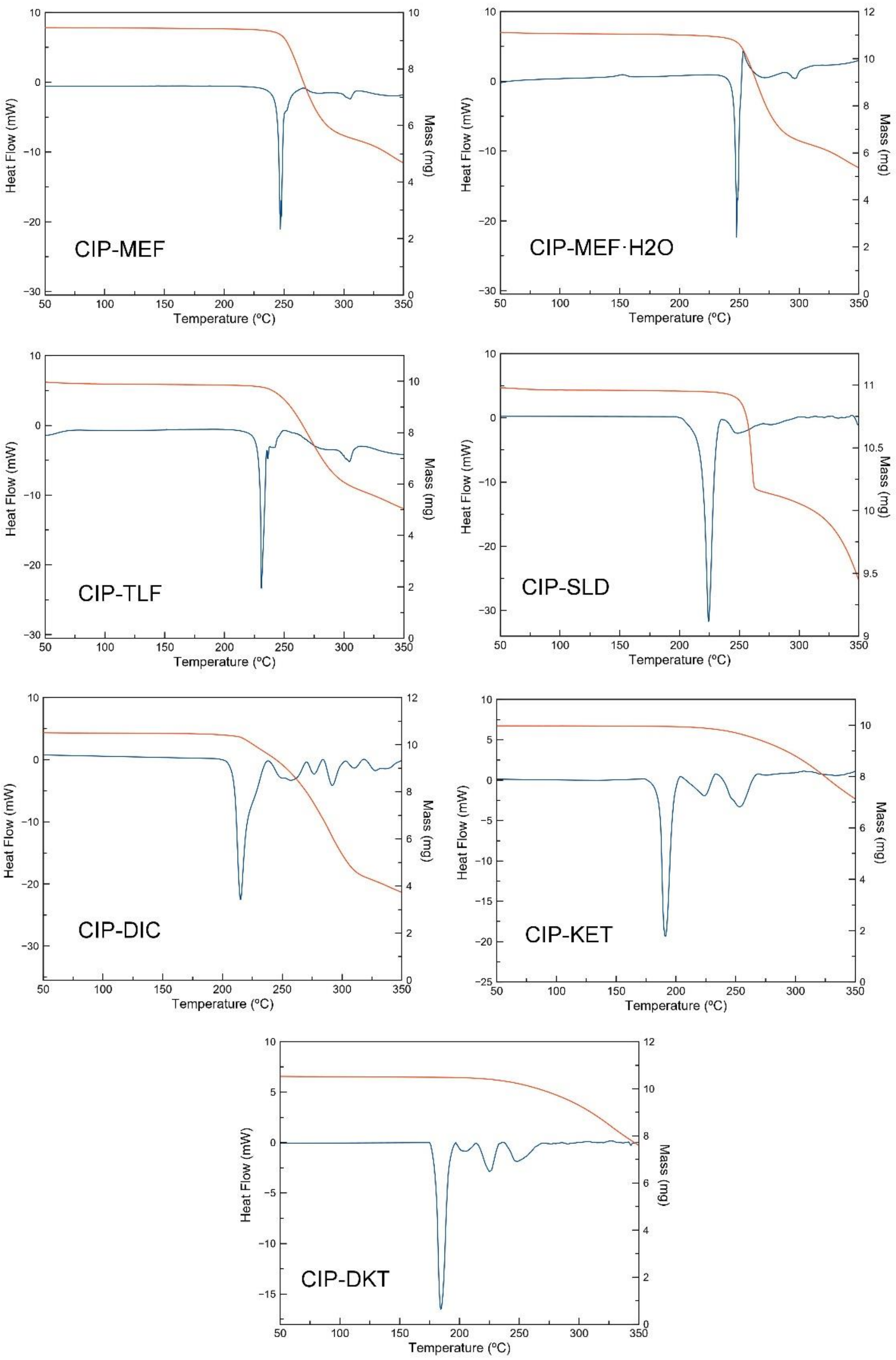

Figure S8. DSC and TG traces of the reported molecular salts.

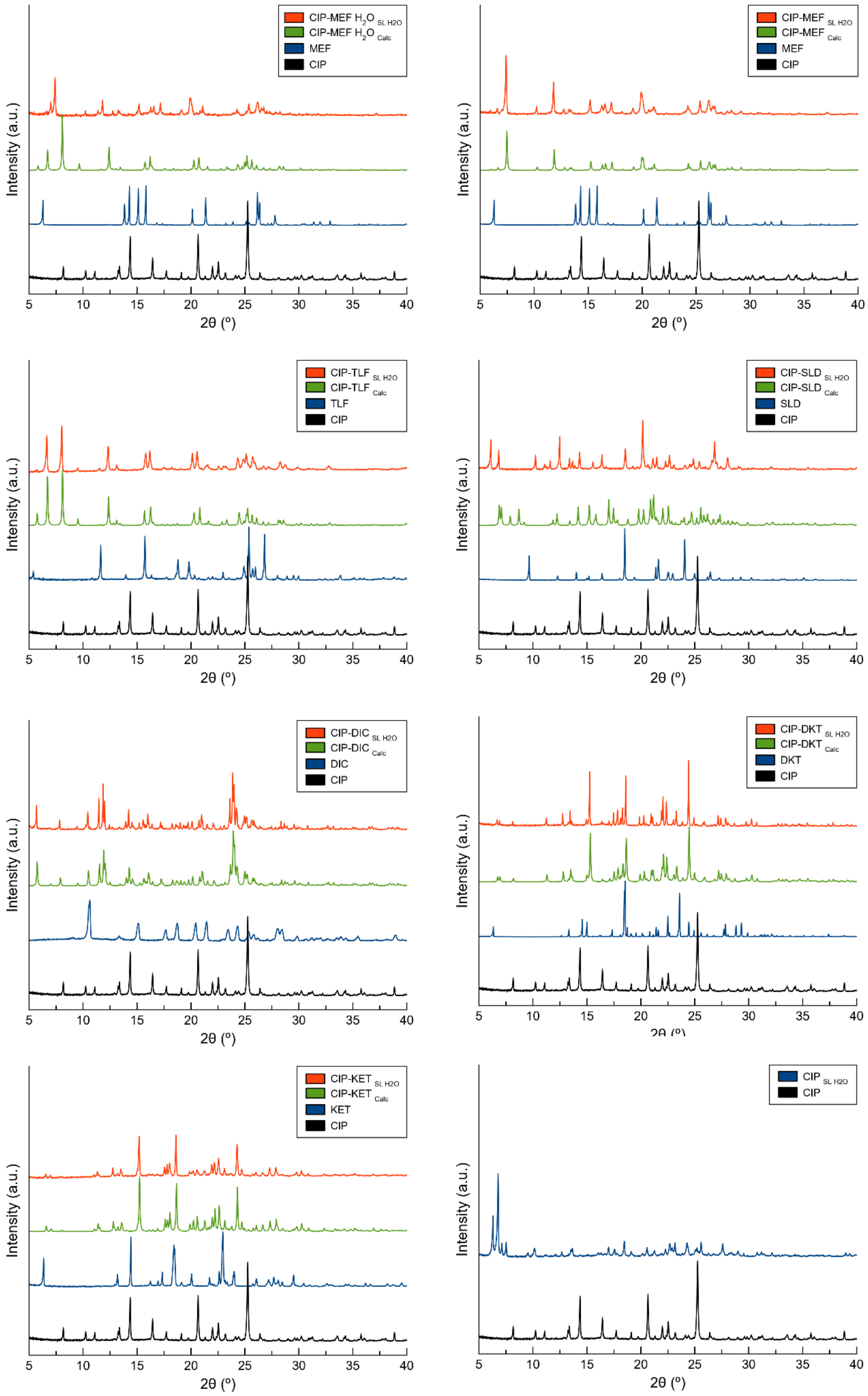

Figure S9. PXRD patterns of the reported molecular salts after aqueous slurrying for 24 hours.

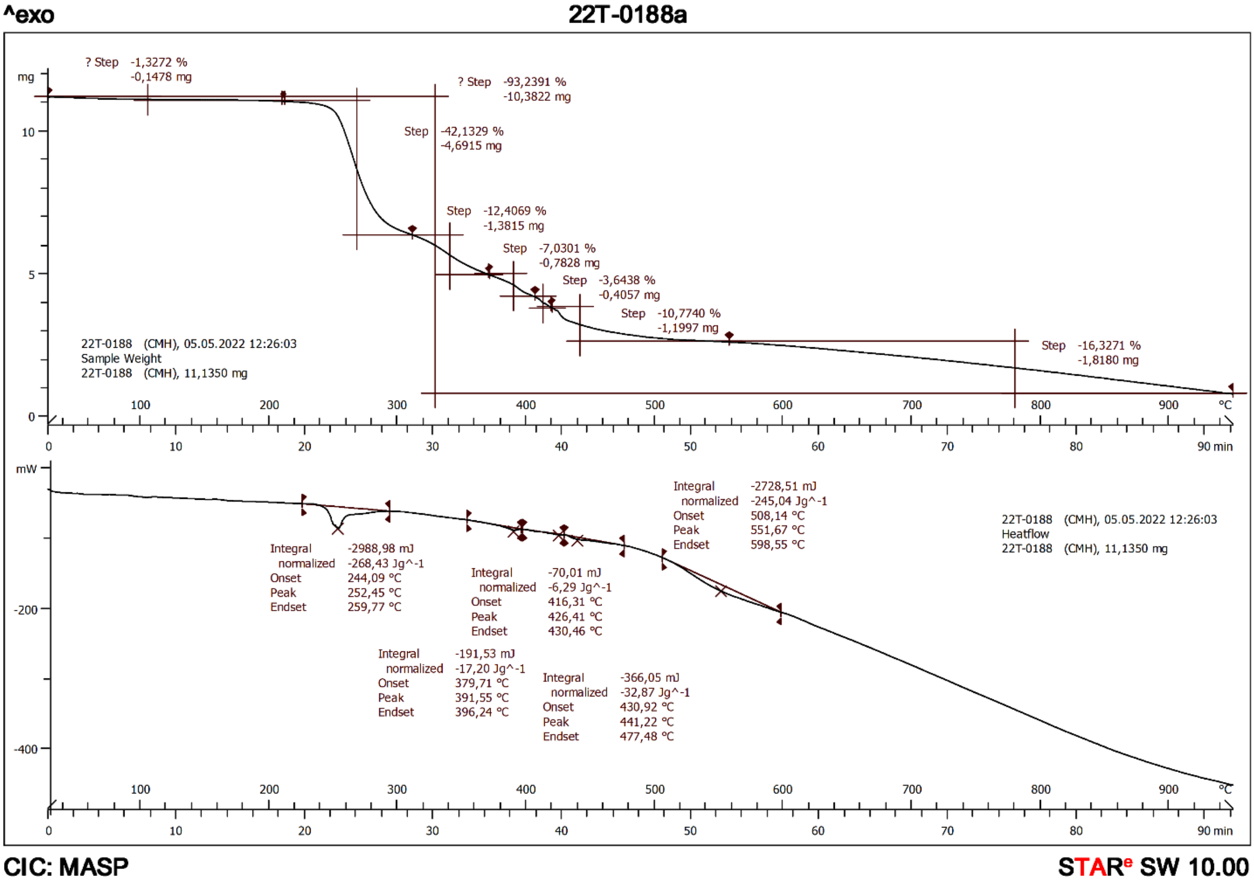

Figure S10. TG analysis of the hydrate salt CIP—MEF·H_2O.

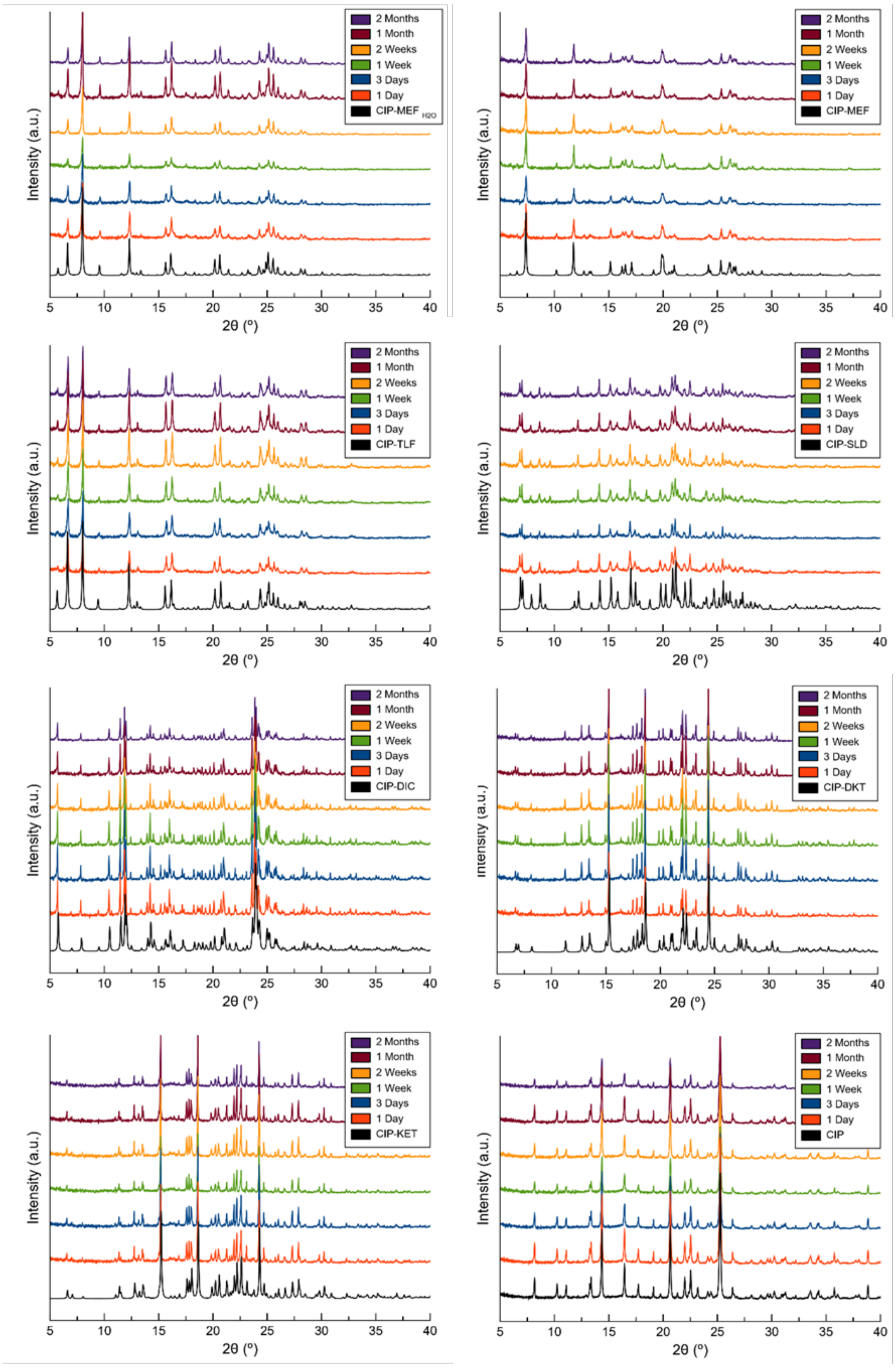

Figure S11. PXRD patterns of reported molecular salts under accelerated ageing conditions for 2 months.

International Journal of
Molecular Sciences

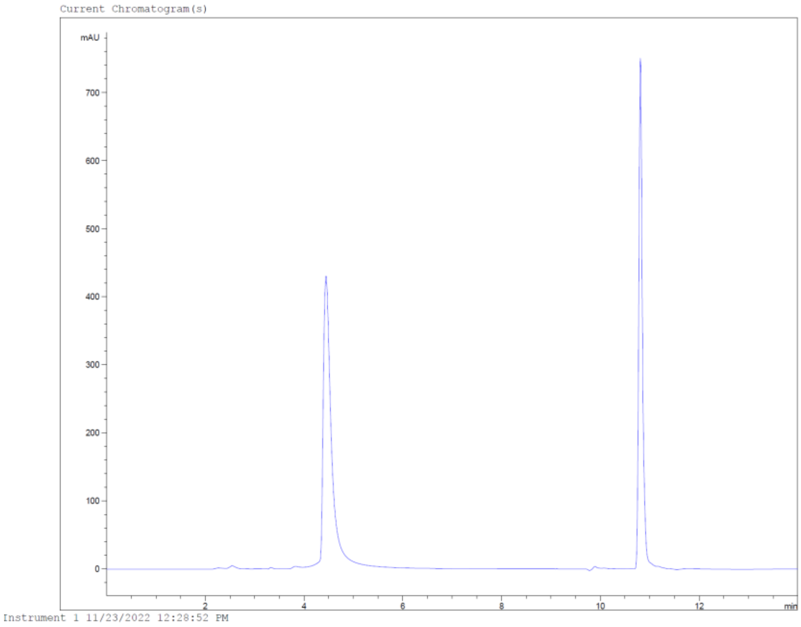

Figure S12. Chromatogram of CIP—DKT salt showing a first peak corresponding to CIP and a second peak corresponding to DKT.

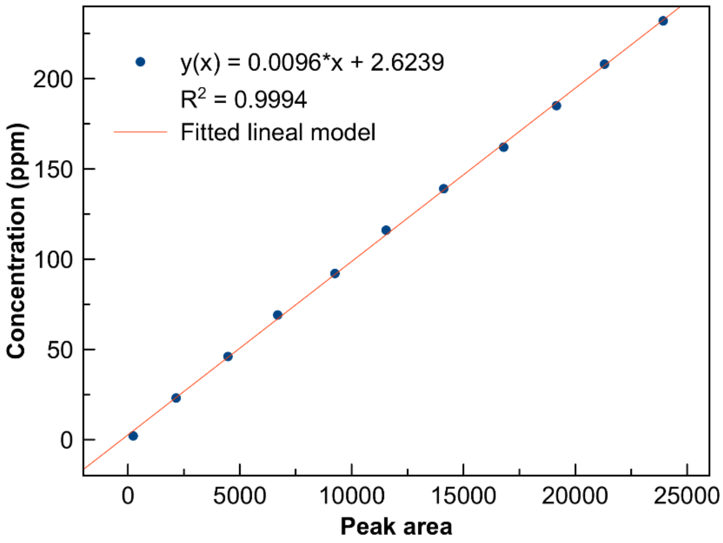

Figure S13. Calibration curve of CIP determined from HPLC data.

International Journal of *Molecular Sciences*

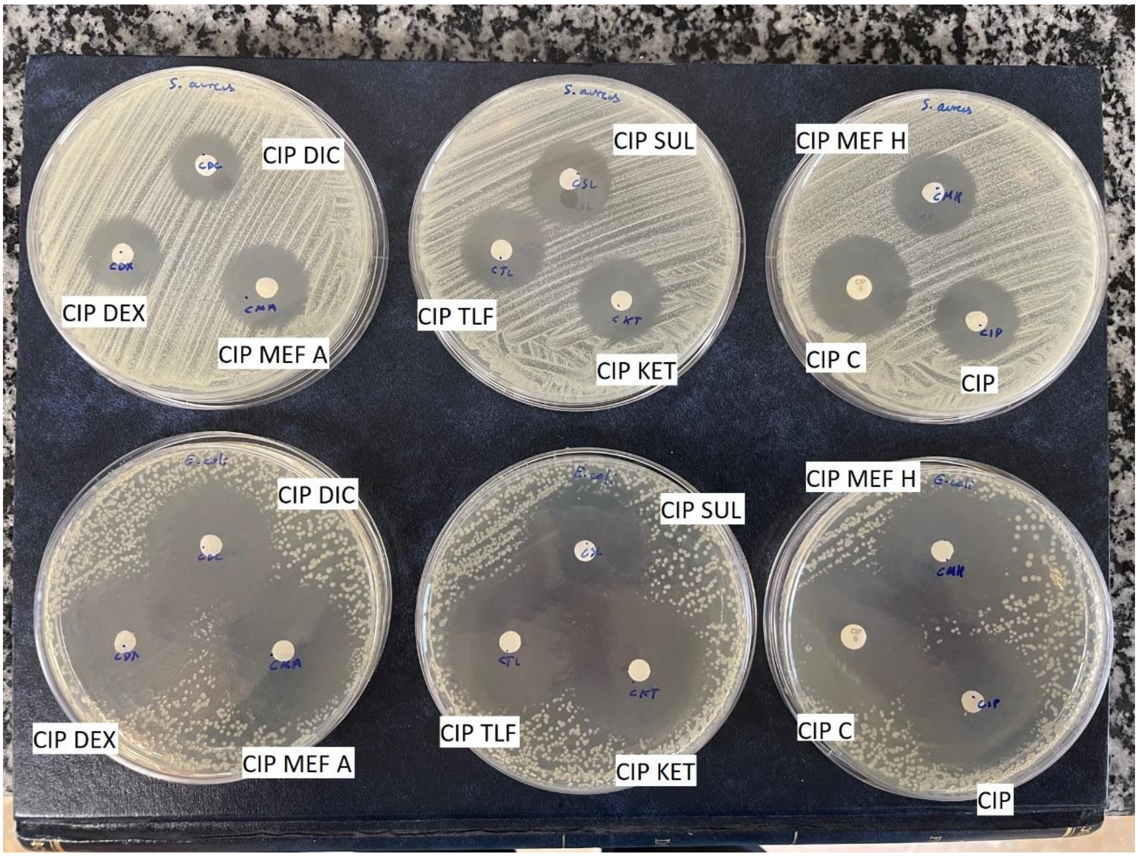

Figure S14. Antimicrobial activity of CIP and CIP phases on TSA agar plates cultured with *Staphylococcus* aureus (ATCC 9144, first row) and *Escherichia coli* (CECT 101, second row).

Table S1. Crystallographic data and structure refinement details of CIP-NSAIDs molecular salts.

Compound name	CIP–MEF	CIP–MEF·H_2O	CIP–TLF	CIP–SLD	CIP–DIC	CIP–KET	CIP–DKT
Formula	$C_{32}H_{33}FN_4O_5$	$C_{32}H_{35}FN_4O_6$	$C_{31}H_{30}ClFN_4O_5$	$C_{37}H_{35}F_2N_3O_6S$	$C_{45}H_{40}Cl_4FN_5O_7$	$C_{33}H_{32}FN_3O_6$	$C_{33}H_{32}FN_3O_6$
Formula weight	572.62	590.64	593.04	687.74	923.62	585.61	585.61
Crystal system	Triclinic	Triclinic	Triclinic	Monoclinic	Triclinic	Triclinic	Triclinic
Space group	P-1	P-1	P-1	$P2_1/n$	P-1	P-1	P1
a/Å	7.0100(3)	7.35750(10)	7.3321(12)	13.2001(12)	7.6759(4)	8.1012(8)	8.2206(2)
b/Å	14.2507(6)	13.7396(2)	13.685(3)	5.5503(5)	15.7770(8)	13.7214(11)	13.6705(4)
c/Å	15.7609(6)	15.4988(3)	15.653(3)	44.790(5)	18.9103(9)	14.0550(13)	13.8361(4)
α/°	70.117(3)	78.3990(10)	78.895(13)	90	95.174(3)	72.384(4)	105.3040(10)
β/°	77.493(3)	82.6850(10)	82.640(14)	93.030(4)	98.141(3)	88.519(5)	106.9440(10)
γ/°	78.337(3)	76.2090(10)	76.679(13)	90	101.593(2)	73.552(5)	91.7470(10)
V/$Å^3$	1431.25(11)	1485.31(4)	1494.0(5)	3276.9(5)	2204.08(19)	1425.1(2)	1424.90(7)
Z	2	2	2	4	2	2	2
Dc/g cm^{-3}	1.329	1.321	1.318	1.394	1.39	1.365	1.365
F(000)	604	624	620	1440	956	616	616
Reflections collected	19699	22333	25965	30549	38733	20166	36525
Unique reflections	5015	4916	5274	5780	7748	4949	9527
Data / restraints / parameters	5015 / 0 / 383	4916 / 0 / 386	5274 / 0 / 382	5780 / 0 / 455	7748 / 0 / 562	4949 / 0 / 390	9527 / 3 / 779
Goodness-of-fit on F^2	0.982	1.095	1.068	0.948	1.063	1.023	1.055
$R1$ (I > 2σ(I))	0.0583	0.0593	0.0730	0.0600	0.053	0.0495	0.0504
w$R2$ (I > 2σ(I))	0.1439	0.1847	0.2424	0.1254	0.1522	0.1296	0.1355

International Journal of *Molecular Sciences*

MDPI

Table S2. Hydrogen bonds for CIP—NSAIDs molecular salts [Å and deg.].

CIP—MEF	D-H···A	d(D-H)	d(H···A)	d(D···A)	<(DHA)
	O(2)-H(2)···O(3)	0.82	1.76	2.528(3)	154.3
	N(3)-H(3A)···O(5)#1	0.89	1.85	2.710(3)	162.0
	N(3)-H(3B)···O(4)	0.89	1.82	2.631(3)	149.8
	C(14)-H(14B)···F(1)	0.97	2.21	2.855(4)	123.2
	N(4)-H(4)···O(5)	0.86	1.93	2.627(3)	137.6
	#1 -x+2,-y,-z+1				
CIP—MEF·H_2O	C(14)-H(14A)···F(1)	0.97	2.21	2.870(3)	124.3
	N(3)-H(3A)···O(4)	0.89	1.80	2.682(2)	171.2
	N(3)-H(3A)···O(5)	0.89	2.60	3.172(3)	122.8
	N(3)-H(3B)···O(5)#1	0.89	1.84	2.680(2)	156.4
	O(2)-H(2)···O(3)	0.82	1.77	2.533(3)	153.8
	N(4)-H(4)···O(4)	0.86	1.93	2.608(2)	134.8
	#1 -x,-y+1,-z				
CIP—TLF	N(4)-H(4)···O(4)	0.86	1.93	2.599(4)	133.6
	O(2)-H(2)···O(3)	0.82	1.81	2.523(5)	145.1
	N(3)-H(3A)···O(5)#1	0.89	1.82	2.659(4)	156.4
	N(3)-H(3B)···O(4)	0.89	1.81	2.690(4)	172.3
	N(3)-H(3B)···O(5)	0.89	2.60	3.182(5)	124.0
	C(14)-H(14B)···F(1)	0.97	2.23	2.880(6)	123.6
	#1 -x,-y,-z+1				
CIP—SLD	O(2)-H(2)···O(3)	0.82	1.75	2.506(4)	153.3
	N(3)-H(3A)···O(4)#1	0.89	2.44	3.078(4)	129.0
	N(3)-H(3A)···O(5)#1	0.89	1.90	2.746(4)	158.9
	N(3)-H(3B)···O(4)	0.89	1.77	2.652(4)	169.1
	C(14)-H(14B)···F(002)	0.97	2.27	2.910(4)	122.4
	#1 x,y+1,z				

International Journal of Molecular Sciences

MDPI

Table S2 (cont.). Hydrogen bonds for CIP—NSAIDs molecular salts [Å and deg.].

CIP—DIC	O(6)-H(6)···O(5)	0.82	1.80	2.605(3)	166.3
	N(5)-H(5A)···O(7)	0.86	2.23	3.026(4)	154.4
	O(1)-H(1)···O(3)	0.82	1.83	2.588(3)	152.9
	N(3)-H(3A)···O(4)	0.89	1.85	2.723(3)	166.2
	N(3)-H(3B)···O(4)#1	0.89	2.40	3.094(3)	135.5
	N(3)-H(3B)···O(5)#1	0.89	2.01	2.876(3)	165.1
	C(14)-H(14A)···F(1)	0.97	2.36	2.984(4)	121.2
	#1 -x+1,-y+1,-z+1				
CIP—KET	O(1)-H(1)···O(3)	0.82	1.75	2.5200(19)	154.8
	N(3)-H(3A)···O(4)#1	0.89	2.39	2.949(2)	121.4
	N(3)-H(3A)···O(5)#1	0.89	1.88	2.769(2)	175.4
	N(3)-H(3B)···O(4)	0.89	1.82	2.693(2)	166.7
	C(14)-H(14A)···F(1)	0.97	2.26	2.906(2)	123.5
	#1 -x+1,-y+1,-z				
CIP—DKT	O(2A)-H(2A)···O(3A)	0.82	1.77	2.526(4)	153.0
	N(3A)-H(3AA)···O(4B)#1	0.89	1.82	2.681(5)	162.0
	N(3A)-H(3AB)···O(4A)	0.89	1.93	2.797(5)	165.7
	N(3A)-H(3AB)···O(5A)	0.89	2.27	2.904(6)	128.0
	C(14A)-H(14B)···F(1A)	0.97	2.25	2.901(5)	123.7
	O(2B)-H(2B)···O(3B)	0.82	1.75	2.519(4)	154.4
	N(3B)-H(3BA)···O(4B)#2	0.89	2.24	2.880(6)	128.6
	N(3B)-H(3BA)···O(5B)#2	0.89	2.04	2.922(6)	168.7
	N(3B)-H(3BB)···O(5A)#3	0.89	1.76	2.623(6)	162.9
	C(14B)-H(14C)···F(1B)	0.97	2.26	2.912(6)	123.3
	#1 x+1,y,z+1 #2 x,y+1,z+1 #3 x-1,y+1,z				

International Journal of Molecular Sciences

MDPI

Table S3. HPLC method parameters.

Parameter	Details
Column	C18 (4.6 mm × 150 mm, 4 μm particle size) Phenomenex
Mobile Phase	MilliQ water with 0.1% formic acid-Acetonitrile (80:20)
Flow Rate	0.5 mL/min
Injected Volume	10 μL
Column Temperature	25 ºC
Sample Temperature	25 ªC
λ_{max}	277 nm
Retention Time	4.5 min
Equation	y(x) = 0.0096*x + 2.6239
Regression Coefficient (R^2)	0.9994
Calibration Range	2– 232 mg/L (ppm)

Experimental procedure for determination of solubility by HPLC analysis.

Instrumentation

A high-performance liquid chromatography (HPLC) with a UV array detection system was used to determine the concetration of CIP. The system was an HPLC Agilent 1200 series and the separation of the target compounds was done using a C18 column (4.6 mm × 150 mm, 4 μm particle size) from Phenomenex, using 0.5 mL/min flow rate and 25°C temperature. UV detection was performed using different wavelengths, one for CIP and another for each NSAID coinciding with their UV maximums of absorbance. At these conditions, the retention time for CIP was 4 min 30 seconds, as can be seen in Figure S1, and from the area under the CIP peak was determined the concentration for the calibration curve. The conditions are summarized in Table S1.

Linearity

The linearity of the developed chromatographic method was evaluated by injecting (10 μL) CIP in the concentration range of 2– 232 mg/L (ppm). The calibration curve was constructed by plotting the peak area under the CIP peak against concentration (ppm). The calibration curve was fitted to a linear model (y(x) = 0.0096*x + 2.6239) with a correlation coefficient (R2) of 0.9994, using the software MagicPlot 3.0.1, where y(x) is the CIP concentration and x is the peak area (Figure S2).

Table S4. Zone of inhibition values of CIP and the reported molecular salts.

Zone diameter values (mm)		
	2.5 µg of compound	
Compounds	*S. aureus*	*E. coli*
CIP	21.12 ± 0.41	33.91 ± 0.36
CIP_c (5 µg)	26.89 ± 0.56	37.77 ± 0.40
CIP—MEF·H_2O	22.07 ± 0.39	33.31 ± 0.49
CIP—MEF	21.20 ± 0.44	34.01 ± 0.36
CIP—TLF	19.98 ± 0.62	33.82 ± 0.37
CIP—SLD	20.03 ± 0.40	31.69 ± 0.60
CIP—DIC	16.34 ± 0.49	32.05 ± 0.53
CIP—KET	22.12 ± 0.53	35.19 ± 0.55
CIP—DKT	18.03 ± 0.42	32.72 ± 0.47

International Journal of
Molecular Sciences

Table S5. Contribution of CIP in each CIP–NSAID molecular salt.

	Molecular weight	*% CIP in the salt*	*μg of CIP in the experiment (2.5 μg total)*
CIP—DKT	585.6	0.57	1.41
CIP—KET	585.6	0.57	1.41
CIP—MEF	572.6	0.58	1.45
CIP—MEF·H_2O	590.6	0.56	1.40
CIP—SLD	687.8	0.47	1.17
CIP—TLF	593.1	0.56	1.40
CIP—DIC	923.6	0.36	0.90
CIP	331.3		2.5

International Journal of
Molecular Sciences

MDPI

Table S6. Final pH values measured during solubility determination by HPLC analysis.

	at pH 1.2	at pH 6.8
CIP—DKT	1.84	5.90
CIP—KET	1.71	6.26
CIP—MEF	2.19	6.98
CIP—TLF	1.51	6.96
CIP—DIC	1.54	6.80
CIP	4.75	4.65
